# Quantum annealing-based route optimization for commercial AGV operating systems in large-scale logistics warehouses

**DOI:** 10.1038/s41598-025-28481-w

**Published:** 2025-12-02

**Authors:** Thinh Nguyen Quang, Kosuke Matsuyama, Keisuke Shimizu, Hiroki Sugano, Eiji Kurimoto, Masahiro Miki, Jumpei Suzuki, Ziyi Chen, Hasitha Muthumala Waidyasooriya, Masanori Hariyama, Masaru Hitomi, Kenta Sawamura, Masayuki Ohzeki

**Affiliations:** 1 Smart Business Solutions Division, Sharp Corporation, Nara, Japan; 2 Graduate School of Information Sciences, Tohoku University, Miyagi, Japan

**Keywords:** Computational science, Mechanical engineering, Computer science

## Abstract

This study proposes a new approach to optimizing the routing of Automated Guided Vehicles (AGVs) in large-scale logistics warehouses using Quantum Annealing. As logistics operations grow, efficient AGV routing becomes critical for ensuring safety, reliability, and throughput, particularly in high-density environments. To address the complexity of real-world systems, we introduce an enhanced cost function that incorporates a real-time priority factor to improve both routing performance and operation safety. In this paper, we present a secure and computationally efficient candidate route generation method, along with an Optimization Problem Clustering technique that decomposes the large problem into smaller, tractable subproblems to reduce computational complexity. The proposed methods are designed to incorporate real-time operational data to ensure collision avoidance, maintain safe operations, and enhance routing efficiency in realistic large-scale warehouse environments. We validate the proposed methods using a state-of-the-art Quantum Annealing machine, benchmarking against both classical and quantum-inspired solvers. Integration with a commercial AGV Operating System (AOS) is demonstrated, enabling seamless connectivity with classical solvers via a local network and with Quantum Annealing and Quantum-inspired solvers via cloud infrastructure. Simulations involving 1000 AGVs show that the proposed route generation method reduces the number of optimization variables by an average of 96% for small-scale to medium-scale systems and 78% for large-scale systems, while also improving route quality. The clustering method reduces the maximum problem size to under 10,000 variables, enabling scalable and safe control of large AGV fleets. Furthermore, a complexity-based problem formulation reduces sampling time by approximately 28.2% compared to conventional size-based approaches for problems ranging from 1000 to 10,000 variables. These results demonstrate the practical viability of Quantum Annealing for managing large-scale AGV Operating Systems and underscore the potential of the proposed methods for advancing logistics optimization in real-world warehouse environments.

## Introduction

### Background

**Figure 1:**
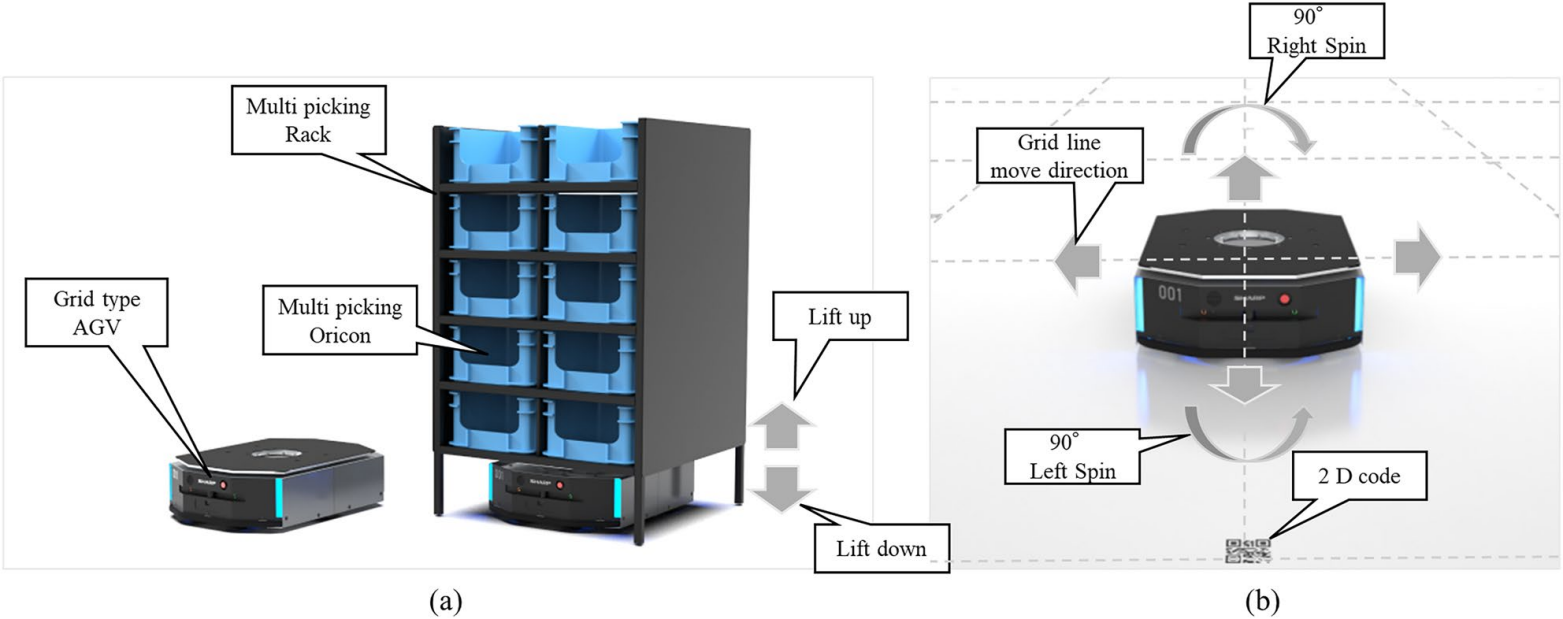
Specifications of the target commercial grid-type AGV^14^. (**a**) shows the specification of an AGV. An AGV of $1,100 \, \textrm{mm} \times 770 \, \textrm{mm} \times 300 \, \textrm{mm}$ in size and supports a payload of 300–1000 kg with multi-type rack depending on the product. (**b**) shows the moving method in a grid-style layout. The AGV reads the 2D code (tag) and recognizes the position. Furthermore, the AGVs could be moved in 6 directions: Forward, back, right, left, $90^{\circ }$ right-spin, and $90^{\circ }$ right-spin. The maximum running speed is 1.67 m/s.

**Figure 2:**
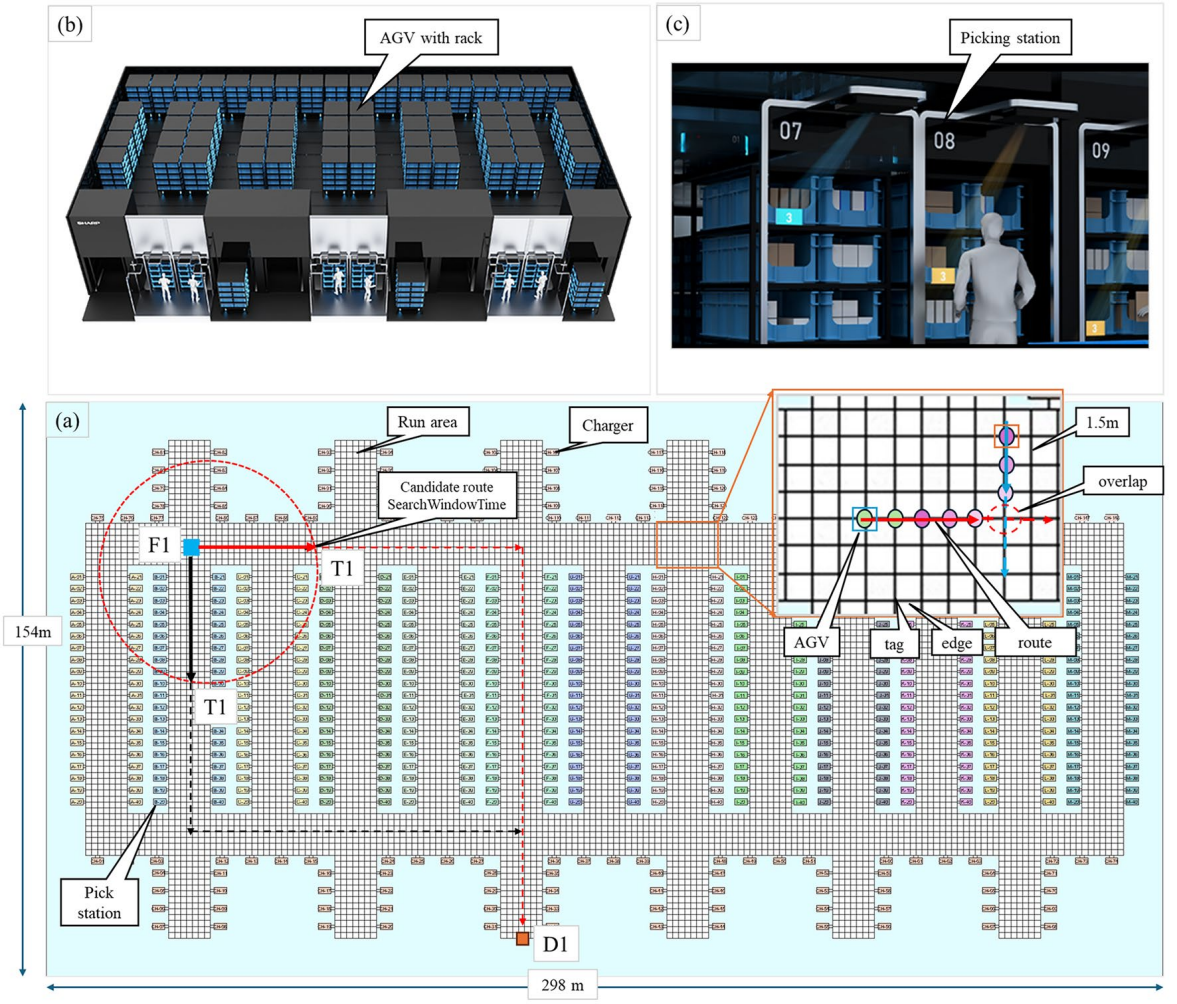
The commercial AGV operating system^14^. (**a**) shows the popular multi-picking operation layout for 1000 AGVs, including tags, edges, routes, and AGVs. The highlighted area represents the travel region, where AGVs and their routes are illustrated. Routes are composed of multiple sequential tags, and the connections between tags, referred to as edges, have a length of approximately 1.5 m to accommodate the AGV size. The route being executed does not overlap, but there is overlap in the candidate route ahead, and route optimization is performed to prevent overlap and collision. This layout spans an area of $154 \, \textrm{m} \times 298 \, \textrm{m}$, supporting the operation of 1000 AGVs with a total of 8,242 tags. (**b**) Illustrates AGVs with picking racks running in high-density conditions. (**c**) Depicts the picking stations utilized in the system.

**Figure 3:**
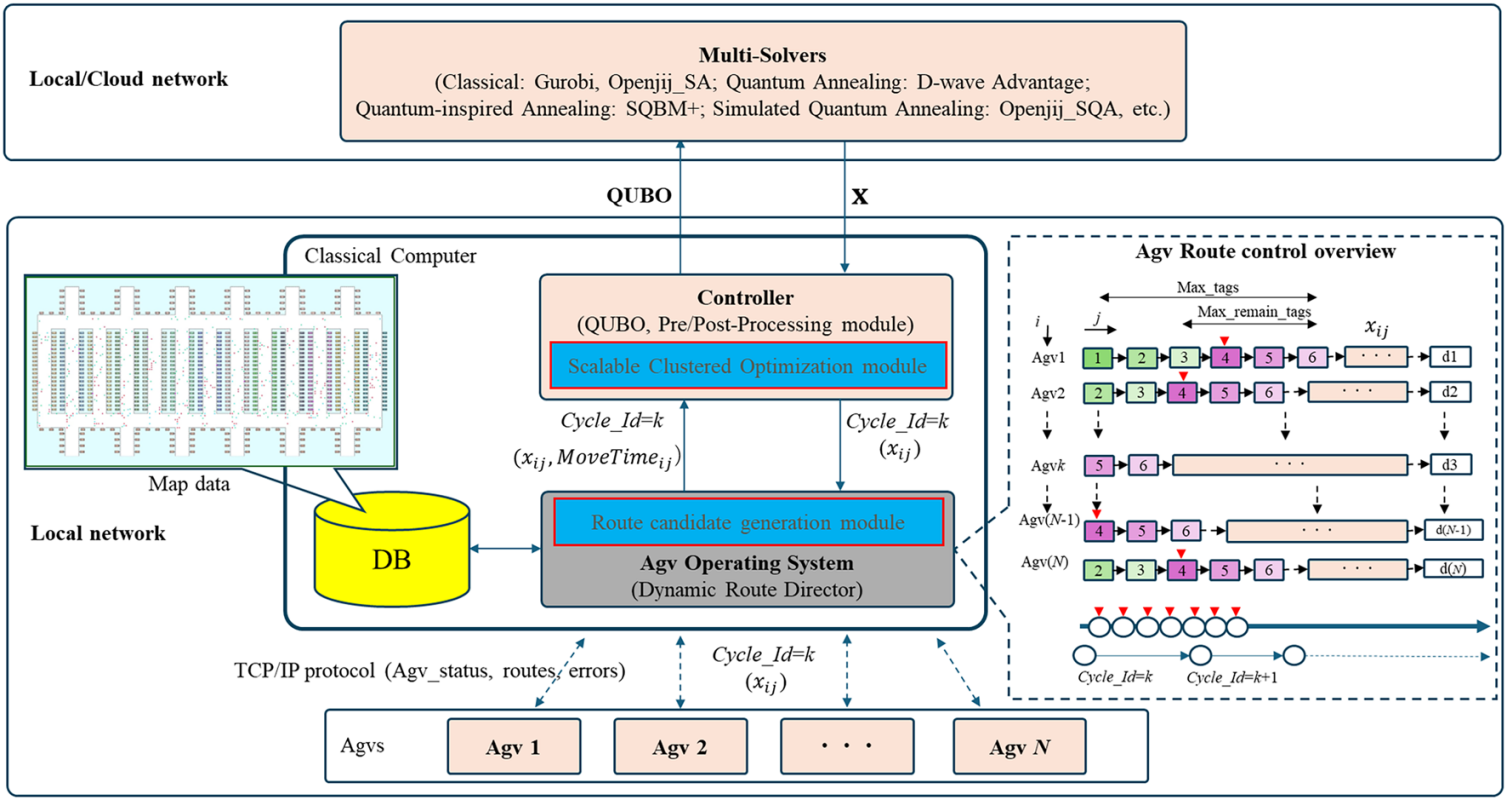
The architecture of the commercial AGV Operating System. AGVs report the status information to the AOS. The AOS, Controller, and Database (DB) run on a classical computer. The Controller connects to solvers such as the D-Wave machine, hybrid solver, and others. The timing of route instructions, route candidates, and route optimization by AOS are shown in the dotted box as the AGV route control overview.

The logistics industry has experienced significant growth in recent years, leading to the expansion of large-scale warehouses designed to manage increasing volumes of goods^1^. As a result, the demand for more efficient and reliable systems for material handling and transportation has intensified. One key technology that has gained traction in these environments is the Automatic Guided Vehicle (AGV) system. AGVs are essential for automating the movement of goods within warehouses, enhancing productivity, and minimizing human intervention. Large-scale warehouses enable better integration of advanced technologies for inventory management, order processing, and transportation, utilizing thousands of automated guided vehicles (AGVs)^2^. As the number of AGVs in operation increases, efficient route planning becomes a critical task. Various routing algorithms have been proposed for multi-AGV systems, incorporating intelligent techniques such as Dijkstra’s algorithm with dynamic priorities ^3^, A* with a queuing mechanism ^4^, Ant colony optimization with an elastic time window ^5^, Genetic algorithms with time windows ^6^, Ant colony optimization with game-theoretic ^7^, and a hybrid genetic-particle swarm algorithm with the dynamic priorities ^8^. Most methods use centralized control, requiring global tracking of AGV positions to avoid conflicts ^9^. However, they are vulnerable to dynamic disturbances, which may require full rerouting ^10^. Therefore, some studies have used reinforcement learning algorithms for AGV route planning ^11–13^. AGVs which are deployed in large-scale systems comprising up to 1000 units, as illustrated in Figs. 1, 2, and 3, are required to meet the highest levels of performance and reliability. They exhibit several key characteristics, outlined below, that make them well-suited for integration into large-scale logistics warehouses:

- **High Precision and Reliability:** As illustrated in Fig. 1a, AGVs are specifically designed for high-precision material handling, minimizing transportation errors. These systems adopt a centralized design, meticulously engineered to deliver consistent operational performance, significantly reducing delays and enhancing the overall reliability of logistics operations. For example, AGVs can lift picking racks weighing between 300 and 1000 kg while achieving a maximum operational speed of 1.67 m/s^14^. The stringent requirements for precision and reliability extend beyond AGV hardware, incorporating the integration of the AGV Operating System (AOS) control method. To ensure efficient and safe operation, AGV routes are strictly managed to prevent overlaps under all circumstances, including error conditions. This design eliminates the need for additional sensors, contributing to streamlined functionality and enhanced operational safety.
- **Advanced Navigation:** AGVs are equipped with sophisticated camera navigation systems, enabling autonomous movement within structured environments. These systems utilize camera recognition to detect 2D codes and operate across four dimensions within a grid layout, as shown in Fig. 1b. All AGVs are managed by the AOS, which monitors real-time status, including position (tags), routes (multiple connected tags), and error information.
- **Dynamic Route Optimization:** The AOS dynamically provides optimal routes for AGVs, as shown in Fig. 2a. By leveraging advanced dynamic route optimization algorithms, the AOS enables AGVs to effectively optimize their routes, minimizing travel time and preventing collisions. This capability significantly enhances overall operational efficiency.
- **Scalability and Flexibility:** The AOS is designed to seamlessly scale, accommodating the growing demands of a facility. Additional vehicles can be integrated into the existing network (racks and picking stations) with minimal reconfiguration, allowing flexible expansion to meet evolving operational requirements, as shown in Fig. 2b,c. Moreover, the system can be applied to multiple Ising machines and is easily adjustable as the number of qubits in quantum annealing machines increases in the near future.

In such complex operational environments, optimizing AGV routes becomes an intricate problem that requires advanced algorithms to manage the flow of hundreds or thousands of vehicles in real time^15^. The necessity for an effective solution is further emphasized by the need to balance operational efficiency such as minimizing travel time, energy consumption, and wear-and-tear of equipment while ensuring safety, reliability, and scalability in the system^15^. In centralized AOS, the Dynamic Route Optimization is one of the key control modules, which requires high-speed optimization considering the real-time operating information. One approach to this challenge is mathematical optimization, which can help identify the best solutions under defined objectives and constraints.

Metaheuristics, as general-purpose algorithms applicable to a broad class of optimization problems, have attracted substantial attention in recent years^16^. Among them, simulated annealing (SA) is a prominent example of Natural Science-based algorithms, characterized as a probabilistic optimization method that aims to locate the global optimum by utilizing thermal fluctuations^17^. These fluctuations enable the system to escape local minima, increasing the likelihood of converging to the global minimum. However, as the number of variables increases, the computational time required to find an optimal solution grows rapidly^18^. Quantum annealing (QA) leverages quantum tunneling to escape local minima, offering potential advantages over classical approaches such as simulated annealing (SA), which rely on thermal fluctuations and are often inefficient for complex landscapes ^19^. QA is a heuristic variant of adiabatic quantum computation (AQC), where adiabatic conditions may not be strictly satisfied, yet it enables finding ground states of Ising models ^20^. Optimization problems are typically formulated either as Ising models with spin variables {-1, 1}, or as Quadratic Unconstrained Binary Optimization (QUBO) problems with binary variables {0, 1}. These representations are mathematically equivalent and interconvertible, making QA a suitable tool for combinatorial optimization. The method is grounded in the adiabatic theorem ^21,22^, which ensures a quantum system remains in its ground state if the Hamiltonian evolves slowly relative to the minimum energy gap. QA applies this principle by gradually transitioning from a simple initial Hamiltonian to a problem-specific one, ideally yielding the ground state as the solution. In QA, quantum fluctuations introduced via a transverse field play a role analogous to thermal fluctuations in SA. As annealing proceeds, the amplitude of quantum fluctuations is reduced, allowing the system to converge to the problem Hamiltonian. Quantum effects such as superposition, tunneling, and entanglement are hypothesized to contribute to performance gains ^23,24^, though their exact role in large systems remains under investigation ^25–28^. Currently, QA hardware (notably D-Wave’s superconducting qubit systems) lacks universality, but enriched annealing paths such as those incorporating non-stoquastic Hamiltonians have shown promise in enhancing performance by mitigating first-order phase transitions ^29–35^. However, trade-offs exist, as these modifications can also reduce the energy gap and robustness. Reverse annealing is a novel extension where the system starts from a classical configuration, allowing local search around candidate solutions and integration with other heuristics (e.g., genetic algorithms) ^36–42^. Practically, QA functions as a black-box optimization tool for minimizing classical Ising models, a task known to be NP-hard ^43^. The Ising formulation is widely used alongside QUBO, and both are readily convertible to other standard representations like graphical models ^44,45^.

Simulation studies have shown that, for specific classes of problems such as the square-lattice Ising model, QA can reach ground states more efficiently than SA^46^. Recent studies have conducted comprehensive benchmarks comparing QA, hybrid quantum-classical solvers, traditional methods such as SA, and mathematical programming solvers like Gurobi. For instance, QA-based hybrid solvers have demonstrated notable strengths in large-scale and densely connected QUBO instances, achieving both higher solution quality and faster runtimes than classical heuristics ^47^. Evaluations on the Max-Cut problem show that hybrid QA and quantum-inspired solvers perform well in small to mid-sized graphs, while classical heuristics are often more effective for larger instances ^48^. Moreover, comparative analyses involving mathematical programming solvers such as Gurobi, while QA solvers remain competitive in combinatorial and binary quadratic problems, they currently lag behind these mature solvers in solving general mixed-integer linear programming (MILP) problems ^49^. In addition, recent benchmarking of QA-inspired heuristic solvers including Toshiba SBM and Fujitsu Digital Annealer has clarified the respective strengths of such solvers across problem types like Max-Cut, NAE-3SAT, and Ising spin models, further highlighting QA’s practical applicability ^50^.

To evaluate the industrial applicability of D-Wave’s quantum annealer, a wide range of practical applications have been proposed across various domains, including finance^38,42,51–55^, traffic management^56–61^, scheduling and logistics^39,62–65^, manufacturing^66–69^, simulation^70–74^, biology^75^, machine learning^76–84^, marketing^85^, and decoding problems^86,87^. The potential of quantum annealing to solve optimization problems with inequality constraints has been particularly enhanced^88^, especially in cases where direct formulation is challenging^89^. Comparative studies have been conducted to benchmark the performance of quantum annealers in solving optimization problems^90^, and quantum effects on cases with multiple optimal solutions have also been explored^91,92^. Furthermore, applications of quantum annealing in machine learning for solving optimization problems have been reported, further demonstrating its expanding potential across various domains^93–98^.

In centralized AGV systems, it is possible to utilize global information such as delivery locations and the current positions of all AGVs for optimized route planning. For instance, a routing algorithm for multiple AGVs has been proposed that utilizes a quantum annealer to determine travel routes between given source destination pairs^66^, aiming to maximize the operational efficiency of AGVs. Further research has focused on improving the objective function and developing optimization methods that incorporate reverse annealing techniques^67^. However, the current limitation of insufficient qubits in existing quantum annealers such as D-Wave Advantage^99^ has hindered its application to large-scale AGV systems.

Addressing the above challenge requires a combination of cutting-edge optimization techniques and computational methods capable of handling large-scale problems. This paper explores such solutions, particularly focusing on the potential application of QA to solve route optimization problems in large-scale AGV systems, marking an important step forward in achieving operational excellence in modern logistics environments.

### Related research

Quantum Annealing is a meta-heuristic optimization technique that can efficiently solve combinatorial optimization problems that are difficult for classical methods. It was proposed by Nishimori and Kagowaki in 1998 as an innovative method to solve complex optimization problems through quantum fluctuations^100^. The Ising model can be readily transformed into several equivalent representations, including the Unconstrained Binary Quadratic Programming (UBQP) formulation ^101^, undirected graphical models ^102^, and the QUBO problem ^44^. Among these, the QUBO and Ising formulations are most used in constructing problems solvable by QA. The QUBO formulation is mathematically equivalent to the Ising model via a straightforward change of basis. Quantum Annealers can be used to optimize many problems that can be represented as a QUBO. It is particularly suited for addressing quadratic unconstrained binary optimization problems, into which many well-known combinatorial problems can be reformulated ^44^. The general QUBO objective function is defined as follows:

1 $$\begin{aligned} \text {Obj}(\textbf{x}, Q) = \textbf{x}^\top Q \textbf{x} = \sum _{i,j=1}^N Q_{ij} x_i x_j, \end{aligned}$$

where $\textbf{x}$ is the binary variable vector, and $Q$ is an ${N \times N}$ symmetric matrix encoding the interaction terms between variables. QUBO has become the de facto standard input format for quantum annealers. Once the problem is defined, it is mapped to a logical graph in which nodes correspond to variables and edges represent interactions between pairs of variables ^103^. While QA natively supports unconstrained binary optimization, real-world applications often involve additional constraints that cannot be directly imposed within the QUBO/Ising framework. Once the Hamiltonian is encoded on the hardware, it is immutable during annealing, and the system will evolve towards a low-energy configuration without explicitly enforcing constraints. To incorporate constraints, they must be embedded within the cost function using penalty terms. This leads to an augmented QUBO objective:

2 $$\begin{aligned} C(\textbf{x}) = \text {Obj}(\textbf{x}, Q) + \sum _{\text {constraints } k} \lambda _k P_k(\textbf{x})^2, \end{aligned}$$

where $P_k(\textbf{x})$ represents a linear constraint function satisfied when $P_k(\textbf{x}) = 0$, and $\lambda _k$ is the associated penalty coefficient. Common examples include one-hot encoding and linear equality constraints. However, care must be taken when choosing penalty weights. If the penalty terms are too small, constraint-violating solutions may appear favorable. Conversely, excessively large penalty values can distort the energy landscape, exacerbating sensitivity to noise in the quantum annealer and possibly masking global optima. It is thus crucial to set penalty factors $\lambda _k$ to the smallest possible values that still enforce constraint validity in all optimal solutions.

We review the studies by Ohzeki et al.^66^, and Haba et al.^67^, which employ a quantum annealer called D-Wave^99^ to solve the AGV routing optimization problem. While these studies focus on relatively routing scenarios involving 10 to 20 AGVs, their fundamental approaches have the potential to contribute significantly to the advancements of the field. In the first study, Ohzeki et al.^66^ propose a cost function aimed at minimizing AGV waiting time, which is based on maximizing AGV movements efficiency. The formulation includes penalty terms to enforce important constraints, such as preventing an AGV from selecting multiple routes and avoiding collisions between AGVs. In the second study, Haba et al.^67^ present a modified QUBO formulation in which the traditional “route distance” is replaced with the concept of “remaining distance”. Specifically, for AGV *i* and route *j*, the remaining distance is defined as the shortest route from the end node of the selected route to the AGV’s destination. The objective is to minimize this remaining distance to improve routing efficiency. The study also introduces the reverse annealing method, which enables obtaining optimal solutions up to ten times faster than classical solvers such as Gurobi for small problem sizes.

In this study, we proposed a new cost function formulation to address the route optimization problem^15^. One major issue with previous studies is related to the QUBO formulation. Specifically, the constraints allow the AGVs to stop while their routes are adjusted for collision avoidance. This results in certain AGVs requiring a long time to find a feasible route. In practical large-scale AGV route control scenarios, travel distances may deviate significantly from travel times due to variations in AGV speeds, idle time caused by constraint violations, and other complex operational factors related to labor. Furthermore, route overlaps can lead to collisions and delays in reprocessing. Another major challenge is the enormous number of variables required to solve large-scale AGV routing problems. A large-scale problem involving 512 AGVs with 16 routes per AGV requires 8,192 variables and operates at a speed of 1.67 m/s^15^. For the larger-scale problems, such as those involving 1000 AGVs, the number of variables can reach up to 30,000. Under real-world operating conditions, the number of required variables increases with problem complexity. Additionally, the total optimization process speed is expected to be much higher. This immense scale far exceeds the capacity of the latest quantum annealer, the D-Wave Advantage (which supports around 5000 qubits)^104^, posing a significant challenge for future advancements in quantum computing.

To address these challenges, this paper proposes a route optimization approach for AGV Operating System, illustrating how Quantum Annealing can be effectively applied to commercial large-scale logistics warehouse operations. This paper presents a route candidate method and a problem clustering method for large-scale route optimization using the real-time operation information. The contributions of this work are summarized as follows:

1. Efficient Route Candidate Generation: In real-world operations, where the route candidate process often becomes a bottleneck, we propose and validate a new approach to generate better route candidates more efficiently. This includes developing and comparing a new route exploration method that accounts for real-time information with the widely used Dijkstra’s algorithm for shortest route computation. By incorporating real-time operational data, the proposed method enhances the quality and efficiency of route candidate generation, reducing bottlenecks and improving overall system performance.
2. Dynamic Problem Clustering: To address the limitations of current quantum annealing machines, which can only handle small-scale problems, we propose a new method for solving large-scale problems. This involves using real-time AGV data, including position, route, error, and job priority information, to compute a complexity index for the overall problem. Clustering techniques are then applied to divide the master problem into multiple subproblems. Each subproblem is dynamically reformulated based on its complexity to match the capabilities of QA or other solvers. The newly formed problems are solved by the quantum annealer machines, allowing for scalable optimization.
3. Commercial System Integrations: We applied the proposal method directly to AGV Operation Systems commercially available in the Japanese market. Validation is conducted to ensure the correct execution of the route optimization method for multiple AGVs in real-world scenarios. We also validated the algorithm by integrating a small-scale commercial AGV system with a commercial AOS and verifying its connectivity to the classical solvers in local network, to the quantum annealing machine, and quantum-inspired annealing machine via a cloud network.
4. Validation Across Multiple Solvers: Various solvers, including classical, hybrid, quantum-inspired annealing, and quantum annealing, are employed to demonstrate the effectiveness of the method under practical constraints and scenarios. A simulation system capable of handling up to 1000 AGV simulators has been developed to demonstrate the effectiveness of the proposed method. In this simulation system, AGV simulators are represented virtually, while all other components utilize the actual applied system, bridging the gap between simulated and real-world operations.

## Methods

This section introduces methods for practical deployment in real-world applications. Large-scale warehouse environments present significant collision risks due to other AGVs reserved with special reason such as error occurs or map design constrains. Autonomous safety mechanisms are essential, even in small-scale systems. In large-scale, fully automated operations, such as unmanned or nighttime settings, additional risks arise from instruction-assigned but unexecuted tags. This study proposes integrating a quantum annealing machine with a commercial AGV Operating System (AOS) for optimized routing in large-scale warehouse environments. We first describe the system architecture connecting the commercial AOS with solvers. Next, we present the optimization problem, which minimizes total process time by balancing the allocation of upcoming and remaining routes. Finally, we introduce candidate route generation and optimization problem clustering methods that incorporate real-time operational data. These methods ensure effective problem-solving by quantum annealers or other Ising-based solvers. The optimization problem is relaxed into a QUBO form and solved heuristically by the quantum annealer.

### The architecture of commercial large-scale AOS

To formulate the optimization problem, we first examine the behavior of a commercial large-scale AOS, as illustrated in Fig. 3. The AOS serves as the central component of the AGV control system, managing AGVs by providing routing instructions over the local network. AGVs connect and report the status information to the AOS via the TCP/IP protocol. The AOS, Controller, and Database (DB) run on a classical computer. The Controller connects to solvers such as the D-Wave machine, hybrid solver, and others. The timing of route instructions, route candidates, and route optimization by AOS are shown in the dotted box as the AGV route control overview. The running routes are monitored by the parameters $Max\_tags$ and $Max\_remain\_tags$, and the route candidates and route optimization are performed from the red $\bigtriangledown$ according to the start of $Cycle\_Id$. For example, AGV1 has a route (1, 2, 3, 4, 5, 6), at $Cycle\_Id$ = *k*, AGV1 is initiated from tag 4 for route candidates and route optimization. The $AGV_{i}$ taking route *j*, represented by $X_{ij}$ which is a candidate in orange marker space for route optimization. Each optimization cycle is identified by $Cycle\_Id$ = *k*, where the target route optimization for AGVs occurs between $Cycle\_Id$ = *k* and $Cycle\_Id$ = $k+1$.The cycle process is repeated until all $AGV_{i}$ arrival at destination ($d_{i}$). As the AGVs move, the AOS computes the next set of routing instructions in real-time based on the execution status of the current route. During each cycle, the AOS generates candidate routes through its Route Candidate Generation module, leveraging map data stored in a central database (DB).

The Problems Clustering module further refines the problem size, adapting it to real-time data and solver specifications. The formulated optimization problems are expressed in the form of QUBO. The route optimization is performed within the local or cloud network, using Ising machines as solvers, including quantum annealing machines, simulated annealing, simulated quantum annealing, or hybrid solvers. Therefore, it is crucial to determine the optimized routes for all AGVs in the subsequent cycle before the completion of their movements in the current cycle.

As the density of AGVs and their travel speeds increase, the likelihood of route overlaps also rises, thereby increasing the risk of collisions. AGVs are subject to various unforeseen disruptions, such as derailments or mechanical failures, which are collectively referred to as “errors” and may occur even during the route optimization process. The locations where these errors occur are marked as “error tags”. When an error occurs in an AGV, it is necessary to secure tags within a radius of x meters from the error occur position. Therefore, the “error tags” are total of tags that are included within a certain safe range around the error position. Furthermore, specific operational constraints, such as the physical size of AGVs or the requirement for continuous movement, referred to as “non-stop tags”, introduce additional complexity to routing decisions. Therefore, it is essential to consider both operational efficiency and overall system safety. Although the route optimization described in Fig. 3 may vary slightly depending on the manufacturer in large-scale centralized control systems, the route optimization module itself is considered to be common. Therefore, while this study has been implemented for a specific commercial system, the proposed method is expected to be applicable to other commercial systems using similar approaches.

### Route optimization cost function

In this study, we have previously proposed a basic cost function^15^. In this paper, we evaluate the performance of all algorithms with the proposed cost functions on a real AOS generated multi-validation problems. We aim to address practical operational conditions in which hundreds of AGVs operate completely unmanned, without reliance on humans or obstacle sensors, ensuring that no route overlaps among AGVs. As explained in the related research section, the shortest travel distance often does not translate directly to the shortest travel time for large-scale AGVs. The reasons are the significantly different speeds of AGVs due to their location and route lengths, the idle time caused by constraints violations, and due to other complex operation situations related to labor. Therefore, the objective function is designed not only to minimize the travel time from the current location to the destination, taking into account the positions of other AGVs and ongoing route information, but also to maximize the subsequent available time for the next assignment, ensuring that it is as long and stable as possible.

The cost function $H(\varvec{\textrm{X}})$ is formulated as shown in Eq. (3), which minimizes the total remaining travel time to the destinations of all AGVs under complex operational conditions. Each parameter is explained as follows. $MoveTimeAll _{i,j}$ denotes the move time required for AGV $i$ to travel from the current position to the destination position using route $j$. $MoveTimeNext _{i,j}$ represents the move time required for AGV $i$ to execute the next optimized route from the current position using route $j$. $X_{i,j}$ is the QUBO variable indicating whether AGV $i$ uses route $j$, taking a value of either 0 or 1. The difference ($MoveTimeAll _{i,j} - MoveTimeNext _{i,j}$) represents the remaining movement time for AGV $i$ when using route $j$. The sum of the remaining movement time for all the routes of all AGVs is the total travel time, which should be minimized. The cost function is defined as

3 $$\begin{aligned} H(\varvec{X})&= \sum _{i}\sum _{j} \left( MoveTimeAll _{i,j} - MoveTimeNext _{i,j} \right) X _{i,j} \nonumber \\&\quad + \lambda _{1} \sum _{i} \left( \sum _{j} X _{i,j} - 1 \right) ^2 \nonumber \\&\quad + \lambda _{2} \sum _{ tag } \left( \sum _{i}\sum _{j} C _{i,j, tag } Pri _{i} X _{i,j} - 1 \right) ^2, \end{aligned}$$

where each constraint is controlled by weight constants $\lambda _{1}$ and $\lambda _{2}$. The values of these constants are determined experimentally. The second and third terms represent penalty terms introduced to avoid infeasible solutions. The first constraint term, $\lambda _{1} \sum _{i} \left( \sum _{j} X _{i,j} - 1 \right) ^2$, ensures that each AGV selects exactly one route from all candidate routes. The second constraint term, $\lambda _{2} \sum _{ tag } \left( \sum _{i}\sum _{j} C _{i,j, tag } Pri _{i} X _{i,j} - 1 \right) ^2$, reaches its minimum value of zero when the corresponding condition is satisfied. This constraint guarantees that collisions among AGV routes are avoided while prioritizing the routing of critical AGVs. $C _{i,j, tag }$ is a binary constant that takes the value of 1 when AGV *i* is using route *j*, and that route passes over the position “$tag$”, otherwise, it takes the value 0. The priority constant $Pri _{i}$ represents the priority value assigned to AGV *i*. This value is determined based on the priority of tasks assigned to the AGV, such as traveling to a charging station, returning to its home position, or waiting at a designated location. In this study, the priority value $Pri _{i}$ is defined as the sum of four key factors: $\text {remain\_tag}(i)$, $\text {waiting\_time}(i)$, $\text {remain\_distance}(i)$, and $\text {destination\_type}(i)$. Specifically, $\text {remain\_tag}(i)$ denotes the number of unexecuted tags remaining in the current route. $\text {waiting\_time}(i)$ represents the duration from the route request time to the present, indicating the waiting time for route optimization. $\text {remain\_distance}(i)$ corresponds to the shortest remaining distance to the destination. Finally, $\text {destination\_type}(i)$ signifies the priority level of the destination, which includes locations such as waiting positions, charging stations, temporary waiting areas, and pick stations. Therefore, the priority constant ensures that AGVs are prioritized appropriately in cases where potential route conflicts arise. As a result, AGVs with critical tasks can proceed while those with lower-priority tasks remain idle, ultimately optimizing overall travel efficiency. The priority constant plays an important role in preventing conflicts when route overlaps occur in practical applications. This factor not only reduces the degree of route overlap but also serves as a practical control policy for potential route overlapping issues arising from heuristic methods.

**Figure 4:**
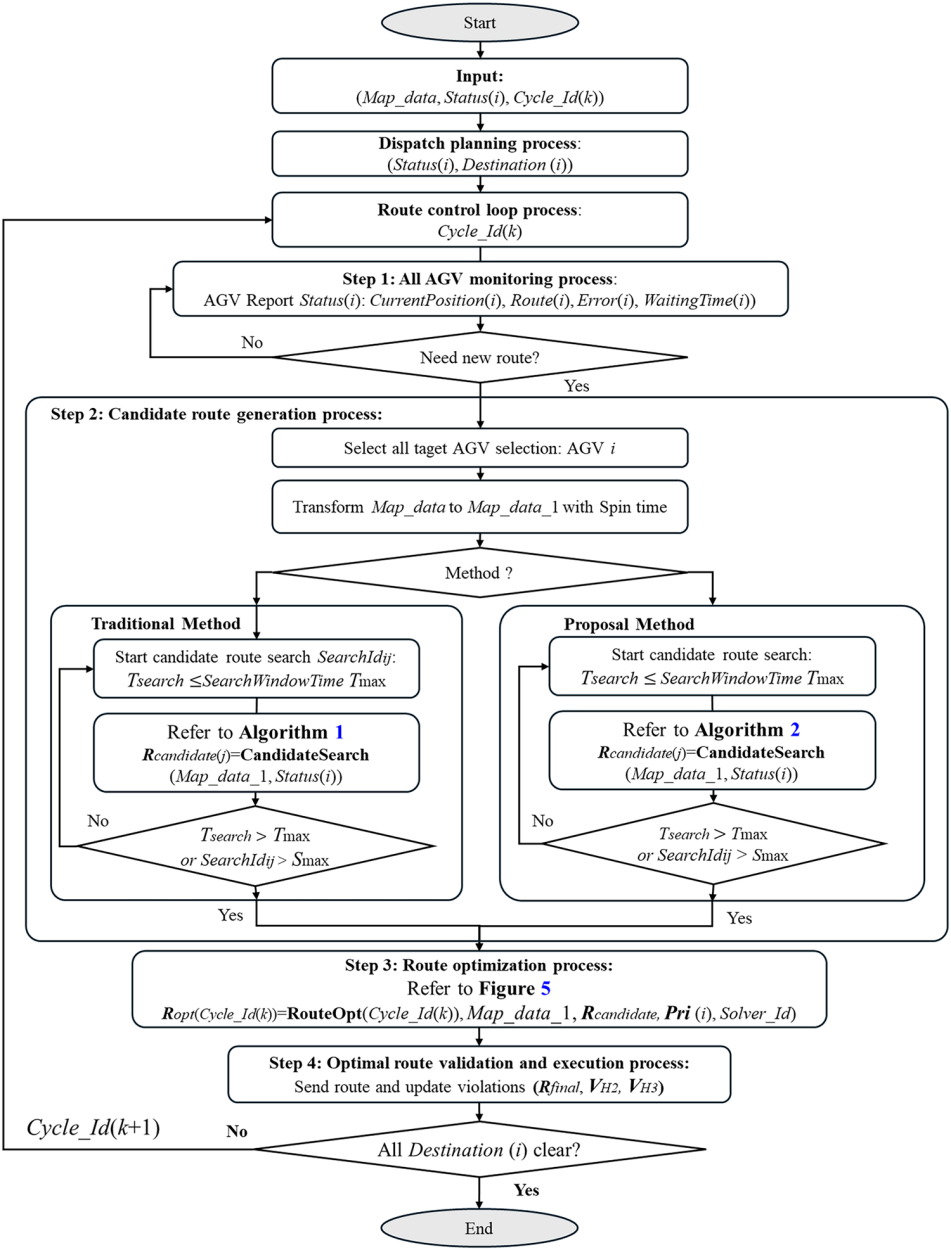
Real-time route optimization method for large-scale AOS. .

### Route control method

The Real-World Large-Scale AGV Route Control process flow (Fig. 4) is designed to efficiently manage route planning and optimization for a large-scale fleet of AGVs in real-time operational environments. The algorithm incorporates real-time operational data to ensure safety, reduce traffic congestion, and improve overall system efficiency. This algorithm consists of some key processes: dispatch planning, AGV status monitoring, route candidate generation, route optimization, optimal route validation and execution, and continuous operation. Each step is vital for ensuring efficient and adaptive AGV navigation. The dispatch plan is the foundation, setting up initial routes for AGVs based on operational priorities, transportation tasks, and system conditions. This step ensures optimal resource allocation, promoting overall system efficiency. For searching the $Cycle\_Id$, there are steps:

**Step 1: AGV monitoring process**. Each AGV reports its status to the AOS, including its position, route, error status, and destination. This real-time data allows the system to adjust AGV routes based on current conditions and disruptions, ensuring continuous optimization.

**Step 2: Candidate route generation process**. Routes are generated based on the AGV’s current position and destination. The following two methods are used to provide multiple route options, making the system more adaptable in complex environments: The traditional method, which relies on historical data without real-time updates (Algorithm 1), and the proposed method, which uses real-time data for dynamic route generation (Algorithm 2).

**Step 3: Route optimization process**. Route candidates are optimized through clustering and quantum annealing. First, the candidates are clustered into smaller subproblems, which are formulated as QUBO models. Quantum annealing is then applied to find the optimal set of routes, improving computational efficiency and solution quality.

**Step 4: Optimal Route Validation and Execution Process**. The optimized routes are validated to ensure compliance with system constraints, such as safety and operational efficiency. The violations are counted in $V _{H2}$ and $V _{H3}$ if any route violates in first and second constraint, respectively. In such cases, the system re-optimizes the routes before dispatching them to the AGVs.

Additionally, the cycle operates dynamically and continuously until all AGVs reach their respective destinations. The AOS executes the Dispatch Planning Process whenever additional requests are detected.

### Route candidate generation method

Algorithm 1 is a traditional method based on Dijkstra’s algorithm, which generates candidate routes by computing the shortest route between the current AGV position and its destination. The key steps include “Route Candidate Target AGV Selection” and “Candidate Route Generation”. Each AGV searches for candidate routes within the “SearchWindowTime” and follows a sequence of processes: “candidate route search”, “penalty weight assignment for special tags”, “sub-route decomposition”, and “movement time estimation”. However, since this method is solely based on the shortest distance calculated by Dijkstra’s algorithm, it does not take into account the presence of other AGVs. Consequently, as the number of AGVs increases, the likelihood of collisions also increases. In real-world commercial applications, this method is suitable for small-scale environments where the map and AOS involve fewer than 50 AGVs and where operational conditions are predictable. The Candidate Route Search process in Step 2 (1)(2)(3)(4) is similar to the process explained in the related research^66^.

Algorithm  2 is the proposed “Route Candidate Generation” which is designated to generate “safe and efficient” route candidates for AGVs in real-time operational environments. Unlike the traditional method, this algorithm accounts for the dynamic and real-time conditions of the AGV fleet to ensure that the generated routes are both “collision-free and optimized for efficiency”. The proposed algorithm consists of key steps, including “Real-Time Data Reporting”, “Route Candidate Target AGV Selection”, and “Route Candidate Generation”. Specifically, in Step 2, by transforming the MAP considering spin time, route information, and real-time factors such as error tags or non-stop tags. The MAP are transformed from $Map\_Data$ to $Map\_Data\_2$ and $Map\_Data\_3$, then Dijkstra’s algorithm can be applied. Furthermore, in Step 3-4, the accuracy of the estimated travel time for newly generated routes is significantly improved. The “SearchWindowTime” and $M$ are parameters to prevent the overtime searching problem for the complex cases. By incorporating real-time operational conditions, the proposed method ensures that the generated routes are both “safe and efficient”, leading to improved resource utilization and reduced optimization time.

As shown in Fig. 3, the AOS receives regular reports from all AGVs as real-time data, such as twice per second, with additional reports triggered by specific events such as start, finish, battery volume, and errors. The frequency of these reports can be adjusted based on communication volume. This allows the AOS to manage real-time data based on the map data (all tags and position information), and the state of all AGVs is rapidly controlled. The candidate route generation module, as described in Algorithm 2, processes this information in each cycle using the proposed method. Specifically, the real-time data, such as “processing tags” which include all the tags of the optimized route, are smaller than the parameter “Max_tags”. The “remaining tags” are those not yet processed, and the “error tags” are the positions where errors occur. The “non-stop tags” prohibit temporary stops as designed by operational constraints. These real-time data are considered when generating candidate routes. This process is achieved by removing inappropriate routes from the map during Step 1 and Step 2 of Algorithm 2. At this stage, safe candidate routes are provided, having fully considered all real-time data, including errors.

**Algorithm 1:**
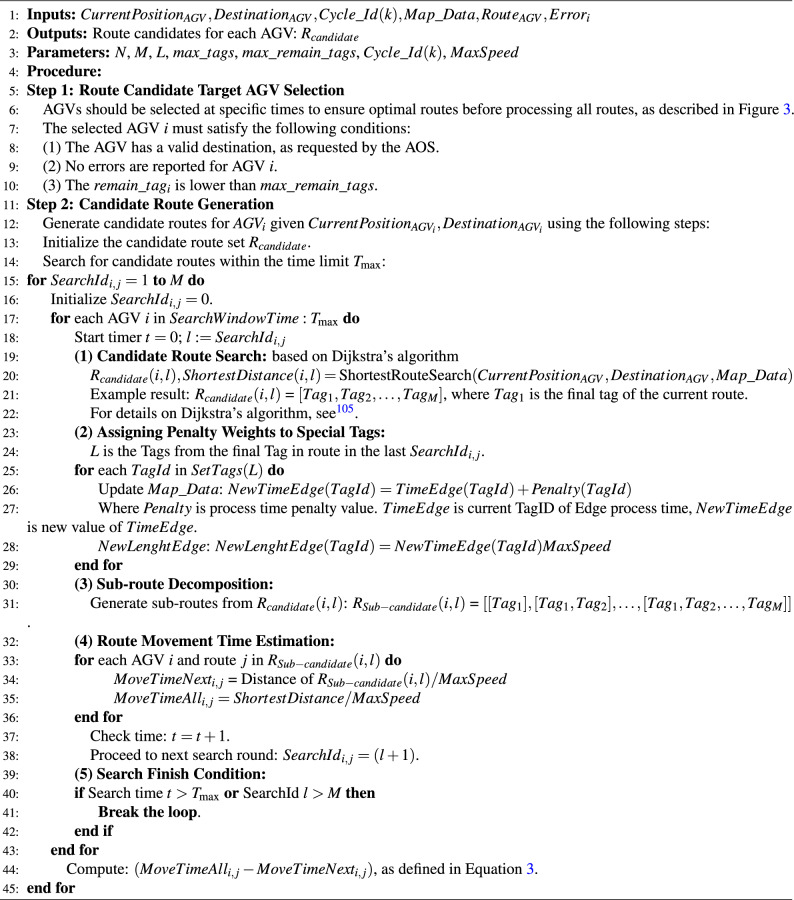
Traditional distance based candidate route generation

**Algorithm 2:**
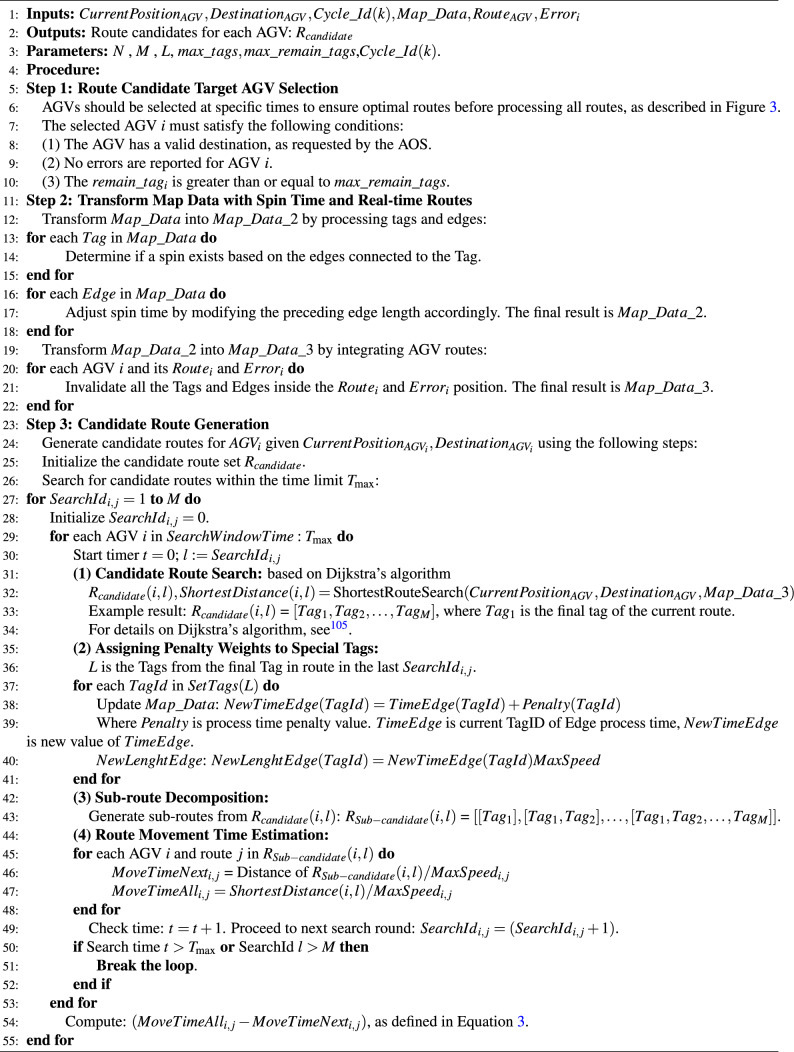
Proposal real-time candidate route generation

**Figure 5:**
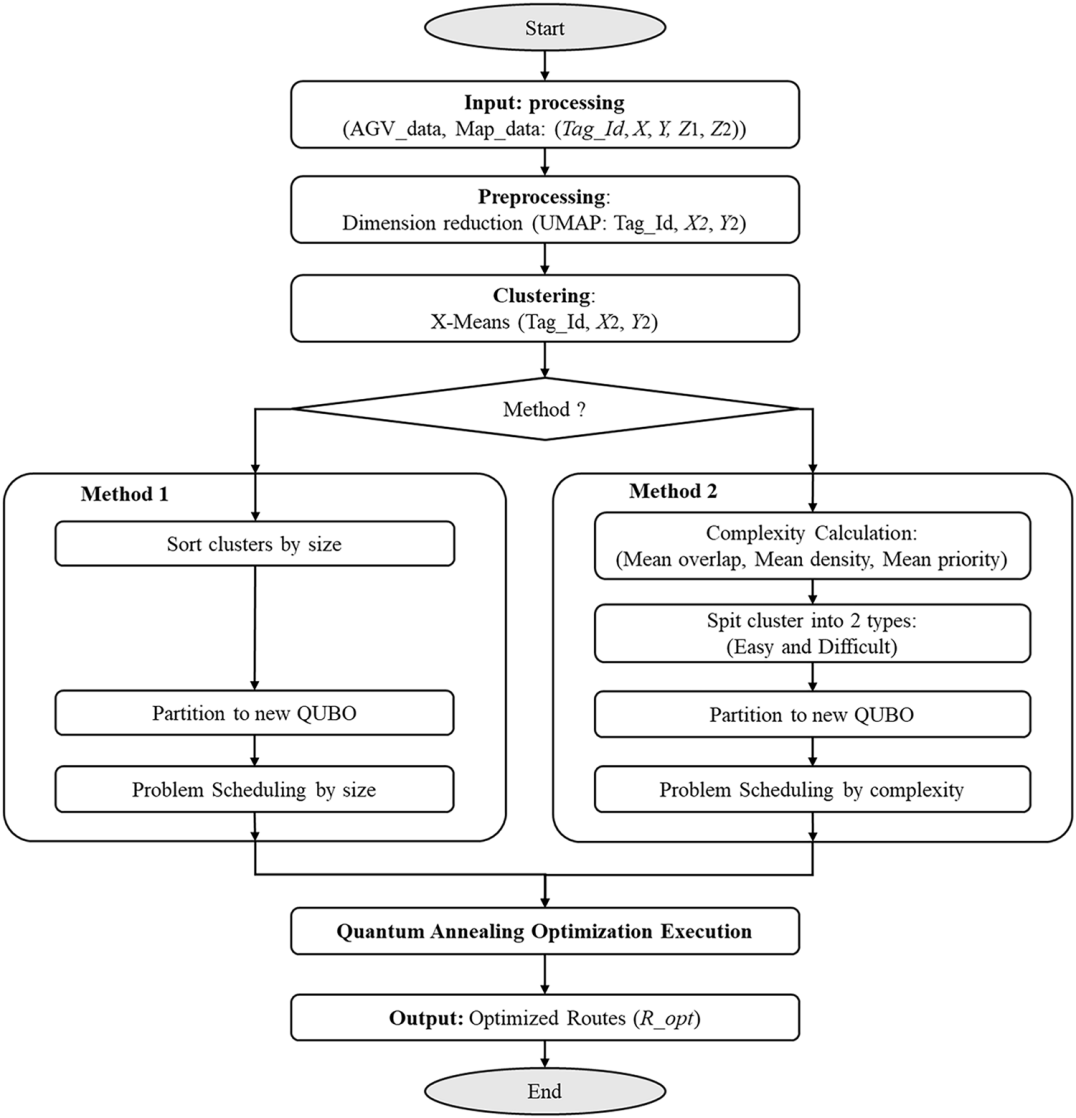
Scalable clustered optimization methods.

### Scalable clustered optimization method

Figure 5 is the Safe and Proper Problem Clustering Algorithm, which partitions large-scale AGV optimization problems into smaller subproblems for efficient processing. The steps are as follows: “Step 1: Map Data Processing”, “Step 2: Route Optimization Problem Clustering”, “Step 3: Problem Partitioning and Scheduling”, and “Step 4: Quantum Annealing Optimization Execution”.

In Step 1, the AGV’s real-time information (location, running information, and candidate routes) is used to calculate the $X$ coordinate, $Y$ coordinate, average tag overlap $Z_1$, and average tag priority $Z_2$ at each tag. Next, coordinate data preprocessing is performed, scaling is performed in the center, and the UMAP method is used to perform high-dimensional reduction from 4D ($X$,$Y$,$Z_1$,$Z_2$) to 2D ($X_2$,$Y_2$) using the Manhattan distance as a metric. In Step 2, the processed MAP ($\textit{Tag\_Id}$,$X_2$,$Y_2$) is used to process clustering using the X-Means method. Here, the X-Means method is an extension of the K-Means method, and the number of clusters is automatically determined. In this research, we investigated both the processing time and the quality of clustering results using the X-means method. The results indicate that the processing time of the X-means method employed in this study is very short, posing no significant issues for real-world applications. Therefore, at this stage, the X-means clustering method was chosen due to its simplicity and ease of implementation. Comparisons with other clustering methods regarding clustering quality will be addressed as a future research challenge, including more recent approaches such as granular-ball DBSCAN for high-dimensional data clustering ^106^, and enhanced t-SNE visualization techniques for complex, large-scale datasets ^107,108^. In Step 3, a new problem is generated from the clustered clusters according to the target solver. Here, we propose two methods. Method 1 adjusts only the problem variable size to fit the solver constraints without considering problem complexity. In contrast, Method 2 evaluates the complexity of each cluster based on real-time information, incorporating factors such as overlap degree, AGV density, and priority. In particular, Method 2 (complexity-Based) evaluates the complexity of each cluster, analyzes the difficult problem (Diff) and the simple problem (Easy) for the entire cluster based on the evaluated complexity, sends the results to the solver in order by simply scaling the complexity in descending order, and performs optimization in Step 4.

In additional, by generating problems based on complexity in Method 2 (2) and Method 2 (3), the problem-solving process can be improved. However, solving only difficult problems may take longer. Since lower-complexity problems can be optimized quickly, sending their optimal routes to AGVs first can help reduce waiting times. On the other hand, incorporating both the route transmission process and problem generation within AGV control increases system complexity. To address this, this study simplifies processing by executing optimization sequentially in ascending order of problem generation after partitioning. Furthermore, since the maximum number of variables can be adjusted as parameters according to the actual solver’s performance, the quality of the optimal solution can be improved. The major advantage of this is that it can be applied as is to current and future solver performance improvements. Therefore, this approach enables efficient optimization by decomposing large-scale problems into manageable subproblems, ensuring scalability and reducing computational overhead.

In the clustered optimization process, large-scale routing problems are partitioned based on the cost function in Eq. (3). The Priority factor is used to allocate overlapping free-space to higher-priority AGVs, effectively reducing collision risks. When a feasible solution is found, safety and efficiency are likely ensured, especially as the solution approaches optimality. As shown in Fig. 5, particularly in Method 2, the system dynamically estimates regional complexity using real-time data such as route overlap, AGV density, and priority levels. Therefore, the formation of smaller, less complex subproblems, making them easier to solve within operational constraints. By integrating this clustering method with the solver, the system proactively prevents collisions from the current cycle into near-future routing.

**Figure 6:**
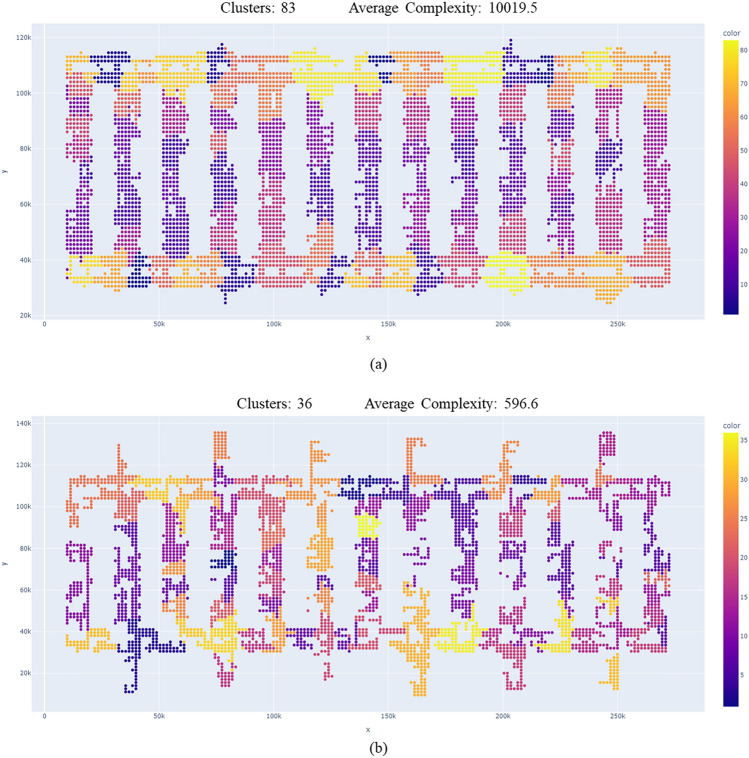
Clustering result samples. (**a**) shows a clustering result using the traditional Candidate Route Generation method with 1000 units, and (**b**) shows a clustering result using the proposal Candidate Route Generation method with 1000 units. While (**a**) and (**b**) are under the same conditions, they produce different clustering results. The proposal method reduces 56.6% of the clusters and 84.1% of average complexity.

Figure 6 illustrates the clustering results using Size-based and Complexity-based methods that are explained in Fig. 5. In (a), the clustering result is obtained using the clustering technique and traditional candidate route generation method with 1000 units, based on position information. In (b), the clustering result is obtained using the proposed candidate route generation method with 1000 units, incorporating all real-time information. The color heatmap represents the complexity of the clusters. Although (a) and (b) are generated under the same conditions as explained in Figs. 1, 2, 3, they yield different clustering results (the clusters and the average complexity). This sample shows the proposed method reduces 56.6% of the clusters and 84.1% of average complexity.

## Results

In this section, we first explain the evaluation conditions, including the experimental system, evaluation settings, and the definition of evaluation metrics, before presenting the results. We also describe the problem instances used for evaluation and the hyperparameter optimization process. In this study, we validate the proposed algorithm by integrating a small-scale commercial AGV system with a commercial AOS and verifying its connectivity to a D-Wave machine via a cloud service. Additionally, we confirm that, even in a local Wi-Fi environment, classical solvers such as OpenJij’s SA^109^, Gurobi^110^, and Gurobi-MILP^67^, as well as quantum-inspired annealing machines, can accurately control the system. The Gurobi was employed using the proposed quadratic cost function. For comparison, the Gurobi-MILP was formulated by converting the problem into a Mixed integer linear programming (MILP) problem formulation based on a previous study^67^, under the assumption that the priority values of all AGVs are set to 1. Due to the high costs associated with deploying a medium-scale and large-scale AGV system, we conduct verification and performance evaluation using the simulation system described in the previous section.

### Conditions

#### Simulation system with commercial AOS

This simulation system is designed to evaluate the effectiveness of the proposed algorithms introduced in Fig. 3. The system consists of the following applications as a commercial system in Japan^14^:

- AOS: A commercial AGV Operating System integrated with a dedicated database. It is responsible for executing route optimization and interacting with the AGV Simulator. The Algorithms 1 and 2 are implemented in the AOS as additional processes. The server specifications include a 10-core 3.10 GHz Intel Xeon w5-2445 CPU, 32GB of DDR4 memory, and a 1 TB SSD.
- Request Plan: A commercial tool used to generate dispatch plans for real-world operations, running on the same AOS.
- Database: A commercial database used in actual operations to facilitate communication between the AGV Simulator and the AGV Operation System. The database is installed on the AOS server. Since suitable public AGV datasets relevant to our research are not available, we used operational scenarios incorporating map data from commercial systems and actual request data from real warehouses throughout the experiments.
- AGV Simulator: A model-based tool that simulates real-world AGVs by considering layout constraints and AGV specifications. This simulator is designed based on actual AGV specifications and real protocols with the AOS, achieving a high simulation accuracy of $\pm 5\%$ in real-world projects. The experimental PC specifications include a 10-core 3.10 GHz Intel Xeon w5-2445 CPU, 32GB of DDR4 memory, and a 1 TB SSD.
- Controller: As introduced in Fig. 3, the controller processes the QUBO and collaborates with the solvers using Algorithm 5. The controller module runs on the same AOS server.
- Solvers: Gurobi, Gurobi-MILP, OpenJij_SA, OpenJij_SQA, and D-Wave machines are used in the experiments. The local solvers run on the AOS server within a local network, while the D-Wave Advantage machine is accessed via a cloud network.

For processing up to 1000 AGVs, only essential information is processed to ensure that the database load remains manageable. This ensures that the simulation system operates at normal speed without significant delays. The simulation system can also be used directly on real AGVs by adjusting the AGV simulator settings to reflect the real AGVs in the system configuration. In this evaluation, we test the algorithms with 2 real AGVs and the commercial system, and with 1000 AGVs in the simulator and commercial system. The proposed method is summarized in Table 1 regarding the experimental conditions. Additionally, the conditions set for each experiment are summarized in Table 2. Since the primary focus of this paper is on addressing large-scale routing optimization problems and facilitating integration with real-world systems, the main solvers considered are QA, Gurobi, SA, and SQA. Within this framework, we investigated the modern Gurobi-MILP solver under both $Pri _{i} = 1$ and $Pri _{i} \ge 1$ conditions. For Gurobi-MILP with $Pri _{i} \ge 1$, we observed scenarios where no feasible solutions satisfying the constraints of the proposed cost function exist. Consequently, the Gurobi-MILP method with $Pri _{i} = 1$ is considered the suitable condition for this approach. A sample of the problem instances used for verification is shown in Table 3.

#### Problem instance sampling

Comparing solvers with the same problem size proved challenging because the problem size varied dynamically due to changing order requirements and the real-time status of the AGVs. Therefore, to effectively and accurately evaluate the algorithms, we generated a set of evaluation route optimization problems using our simulation system, along with an Openjij’s SA solver. In each cycle, the solver is provided with an evaluation problem in the form of QUBO data, which is then output as a CSV file. We generate problems of varying sizes (small, medium, large), the following route candidate generation methods were introduced in Algorithm 1 and in Algorithm 2. The problem sizes are determined based on the number of variables. Using Algorithm 1, instances with over 10,000 variables were recorded, whereas Algorithm 2 generated instances exceeding 30,000 variables. We first randomly selected 190 instances from the problems generated by continuous simulations. Then, we further selected 34 representative instances at random, a subset of which is summarized in Table 3. These problems were then used to assess the performance of the proposed algorithm.

#### Hyperparameter optimization

In solving the route optimization problem, it is necessary to adjust the weights of the constraint conditions, denoted as $\lambda _1$ and $\lambda _2$, which are hyperparameters, as shown in Eq. (3). To optimize these hyperparameters, we use Optuna, a tool for hyperparameter optimization ^111^. Optuna optimizes hyperparameters by defining an objective function, called the Score, which evaluates the performance of different hyperparameter settings. The objective function is defined as follows:

4 $$\begin{aligned} \text {Score} = \sum \left( \frac{N_o}{N_c} - \frac{\sum N_v}{N_c N_R} \right) , \end{aligned}$$

where $N_o$ is the number of samples that satisfy the constraints and achieve the optimal solution with the same energy value, $N_c$ is the total number of samples, $N_v$ is the number of violation routes, and $N_R$ is the total number of candidate routes. The first evaluation metric is the success rate, while the second is the constraint violation rate. The objective function Score primarily aims to maximize the success rate. If the success rate is zero, the objective shifts to minimizing the constraint violation rate. Thus, during the hyperparameter optimization process, the Score is used as the objective function to evaluate performance. We randomly selected five problems generated using the existing route candidate method and five problems generated using the proposed route candidate method and conducted an Optuna optimization experiment. Through this process, we determined the optimal hyperparameter values for $\lambda _1$ and $\lambda _2$ as defined in Eq. (3). The experiments were conducted using Optuna’s default sampler, the Tree-structured Parzen Estimator (TPE) algorithm^112^, and the obtained results are summarized in Table 4, and be applied to performance evaluation experimentation.

**Table 1:** Methods overview.

No	Method	Description	Literature
1	DCG	Traditional Distance based Candidate Route Generation method.	Algorithm 1
2	RCG	Proposal Real-time data based Candidate Route Generation method.	Algorithm 2
3	NCO	Non Clustered Optimization method.	OFF Figure 5
4	SCO	Size-based Clustered Optimization method.	Figure 5
5	CCO	Proposal Complexity-based Clustered Optimization method.	Figure 5
6	DCG+NCO	Traditional Distance based Candidate Route Generation method.	Algorithm 1,OFF Figure 5
7	RCG+NCO	Proposal Real-time data based Candidate Route Generation method.	Algorithm 2,OFF Figure 5
8	DCG+CCO	Traditional Distance based Candidate Route Generation method.	Algorithm 1,ON Figure 5
9	RCG+CCO	All proposal methods.	Algorithm 2,ON Figure 5

The candidate route generation methods are DCG and RCG, while the clustered optimization methods are SCO and CCO.

**Table 2:** Evaluation conditions setting.

**Benchmark evaluation conditions settings**			
Parameter	Gurobi or Gurobi-MILP	Openjij_SA	QA
AGV Numbers (*N*)	10,20,50,100,200,...,1000	10,20,50,100,200,...,1000	10,20,50,100,200,...,1000
Variables	10,100,500,1000,...,30,000	10,100,500,1000,...,30,000	10,100,500,1000,...,30,000
Candidate Route Method	DCG/RCG	DCG/RCG	DCG/RCG
Clustering Method	NCO/SCO/CCO	NCO/SCO/CCO	NCO/SCO/CCO
Max_tags (tags)	18	18	18
Max_remain_tags (tags)	10	10	10
Search_Time (ms)	$3 \times 10^8$	$3 \times 10^8$	$3 \times 10^8$
Max_Candidate_Search	100	100	100
Penalty_On_Tag	True/False	True/False	True/False
Used_Tag_Penalty	15	15	15
N_Samples	1000	1000	1000
Num_Sweeps	100	100	100
Annealing time ($\mu s$)	-	-	20
Number of experiments	1	5	5/15
$\lambda _1$	From Table 4	From Table 4	From Table 4
$\lambda _2$	From Table 4	From Table 4	From Table 4

The solver SA is selected in the problem generation step. All solvers are used in the evaluation experiment with the settings shown above. The number of experimental repetitions for both QA_RCG+CCO and QA_DCG+CCO is 15, respectively.

**Table 3:** Sample evaluation problem instances.

InstanceID	Type	AGVs	Variables
qubo1000003	Type 2	10	31
qubo1000008	Type 2	20	31
qubo1000013	Type 2	50	350
...	...	...	...
qubo1000092	Type 2	1000	4425
qubo1000103	Type 1	10	31
qubo1000108	Type 1	20	36
qubo1000113	Type 1	50	390
...	...	...	...
qubo1000190	Type 1	1000	35,378

A total of 190 instances were randomly selected from the problems generated through continuous simulations.

**Table 4:** Hyperparameter optimization using OpenJij SQA with Optuna.

Optimization settings				Results by OpenJij SQA				
Problem	$\lambda _1$ range	$\lambda _2$ range	Trials	$\lambda _1$	$\lambda _2$	Success rate	Violation rate	Score
DCG (5 size problems)	0–1000	0–1000	1000	53.003	0.235	0.98	0.063	0.917
RCG (5 size problems)	0–1000	0–1000	1000	21.226	90.06	0.66	0.037	0.623

The table presents the optimization parameter settings and optimal hyperparameters, along with objective function values such as accuracy, constraint violation rate, and overall score.

**Table 5:** Experimental results on real AGV and AOS.

Method	Solver name	Tasks per AGV	Max variables	Total process time (ms)	Mean cycle time in solver (ms)	Completed tasks
DCG+NCO	Gurobi	10	17	378,467	4.5	100%
RCG+NCO	Gurobi	10	13	328,944	2.5	100%
DCG+NCO	Openjij_SA	10	16	344,467	9.5	100%
RCG+NCO	Openjij_SA	10	12	309,577	8.6	100%
DCG+NCO	SQBM+	10	16	394,101	1537.7	100%
RCG+NCO	SQBM+	10	10	336,118	1630.4	100%
DCG+NCO	D-wave Advantage 4.1	10	16	424,846	2492.8	100%
RCG+NCO	D-wave Advantage 4.1	10	13	374,083	2189.7	100%

The basic solvers of classical, quantum-inspired annealing, and quantum annealing are tested to verify the performance of the proposed architecture. As shown in the results, small-scale AGVs can achieve route optimization within 2.5 s using both classical and quantum annealing solvers. Locally executed solvers, such as Gurobi and OpenJij_SA, obtain optimal solutions quickly, while cloud-based solvers like SQBM+ and D-Wave machines take longer due to solver characteristics and network latency. The proposed method (RCG+NCO) further improves optimization efficiency by considering real-time information and reducing the number of required qubits.

#### Continuous random optimization experiments

In the continuous experiments on random optimization problems, we conduct experiments using the same simulation system and the same dispatch order data. The experimental setup conditions are summarized in Table 1. Since this experiment reflects real-world operational conditions, the problems are generated dynamically based on the situation. Therefore, both the route candidates and the route optimization problems are expected to exhibit a high degree of randomness. The number of experimental repetitions is indicated as “Number of experiments“ in Table 2. Since Gurobi is designed to obtain the global solution, only a single experimental run was conducted for this method. In contrast, the SA and QA solvers were each tested 5 times. Notably, for the QA_RCG+CCO and QA_DCG+CCO methods, the number of runs was tripled to 15 times to account for variability in heuristic optimization results.

**Figure 7:**
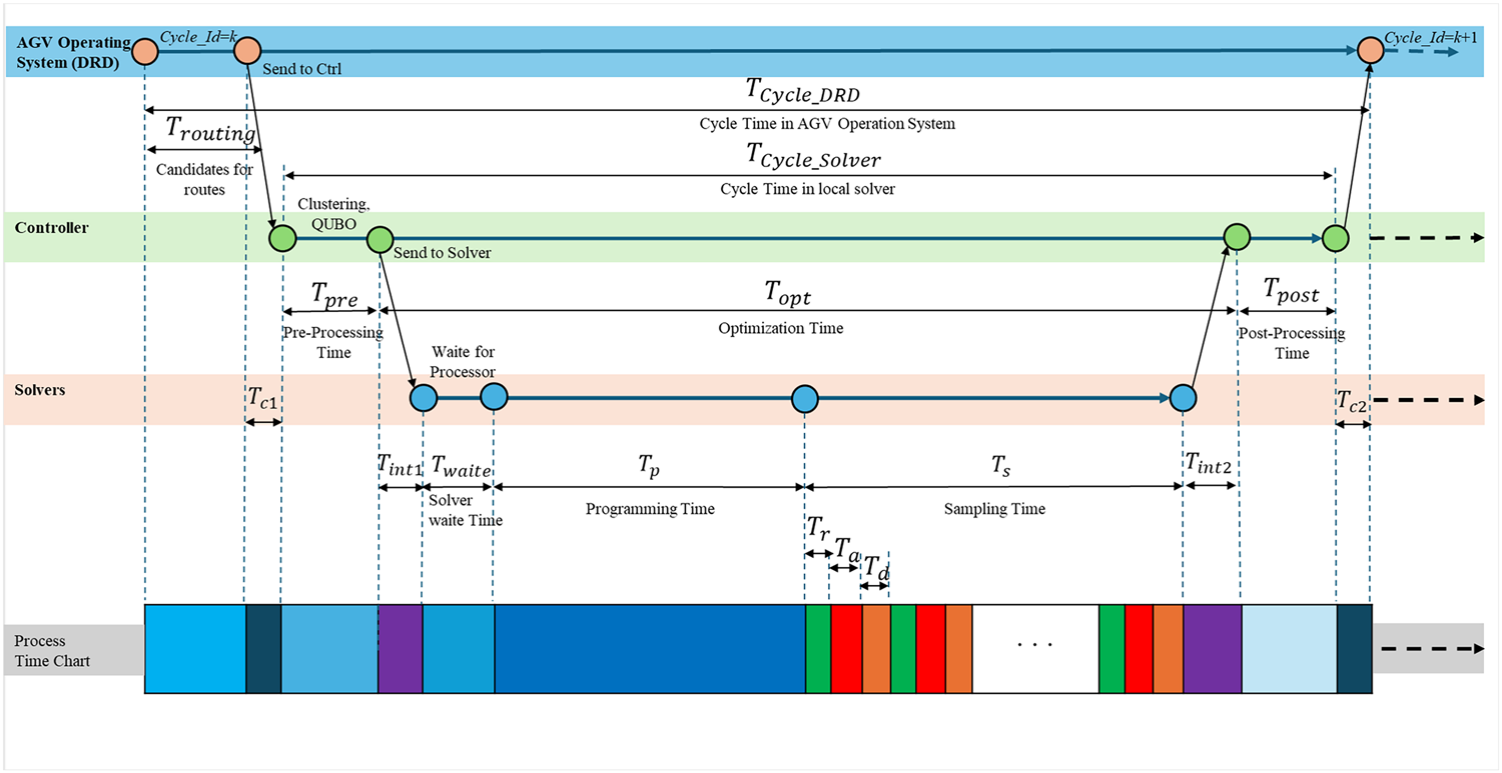
The route optimization process time chart. The processing times between $Cycle\_Id$ = *k* and $Cycle\_Id$ = $k+1$ is shown on the bottom gray bar, which remains nearly the same across all solvers. There are minor differences depending on whether the solver operates in a local network or a cloud network. The processing time is divided into three levels: the AOS based on (Dynamic Route Director: DRD) as the First system level ($T_{\text {Cycle\_DRD}}$), the Controller as the second level ($T_{\text {Cycle\_Solver}}$), and the Solvers the third level ($T_s$).

### Experimentations

#### Experiment on a real AGV and a commercial AOS

In the demonstration experiment, the proposed method was verified using two real AGVs to evaluate whether the quantum annealing-based approach could be correctly integrated into a commercial AOS. This small-scale AGV experiment was not intended to demonstrate scalability. However, scalability was evaluated in subsequent benchmark optimization performance experiments using a simulation system. This system combines the same integrated commercial platform with up to 1000 AGV simulators that employ the protocols provided by the AGV manufacturer. Communication between the AGVs and AOS was carried out via TCP/IP, as shown in Fig. 3, with real-time information such as position data being reported. For each Cycle_ID, the AOS collaborated with multi types of solvers such as classical (Gurobi, SA), quantum-inspired annealing (SQA, SQBM+), and quantum annealing (QA) to generate route candidates and perform optimization. The optimized route was then transmitted to the actual AGVs. This process was repeated continuously and the results are summarized in Table 5.

#### Benchmark optimization performance evaluation

In the performance evaluation experiments, we apply multiple solvers with the proposed method to the same set of pre-generated optimization problems, selected from predefined problem instances rather than random ones, to ensure a quantitative comparison of solver performance. The experimental conditions are summarized in Table 1, and Table 2. Each experiment is conducted five times using the same procedure.

### Evaluation metrics

In this paper, a series of experiments were conducted to demonstrate the effectiveness of the proposed method, including the generation of problem instances for verification, hyperparameter Optimization, verification of route candidate methods, validation of problem clustering methods, and quantitative evaluation of the proposed method. In each experiment, the evaluation metrics used were processing time, optimization results (number of routes and number of route violations), processing capacity, time to obtain the optimal solution, and residual energy. These evaluation metrics are defined as follows.

#### Processing times

The basic processing times are defined according to the sequence of processes shown in Fig. 7. Specifically, the cycle time from the perspective of the AOS is denoted as $T_{\text {Cycle\_DRD}}$ including the Candidate route generation denoted as $T_{\text {routing}}$, while the cycle time from the perspective of the Controller is $T_{\text {Cycle\_Solver}}$. The solver’s sampling time is represented by $T_s$, Clustering time is represented by $T_{clustering}$, and the Pre-Processing and Post-Processing times are $T_{\text {Pre}}$ and $T_{\text {Post}}$, respectively. The impact of sampling time on the accuracy of the solver and the proposed method, as well as the impact of external factors such as preprocessing time and postprocessing time, are quantitatively investigated. If the clustering method is applied and Fig. 5 is used, the Controller sequentially sends each cluster’s QUBO to the solver. As a result,$T_{\text {opt}}$is processed repeatedly based on the number of clusters.

#### Optimal routes

The total number of optimal routes is $R_{opt}(Cycle\_Id(k))$ as defined in Fig. 4. The total number of violations, $V_{Total}$, is given by:

5 $$\begin{aligned} V_{Total} = V_{H2} + V_{H3}, \end{aligned}$$

where $V_{H2}$ and $V_{H3}$ are defined at the first and second constrain in Eq. (3), respectively. The violations are measured in Step 4 in Fig. 4.

#### Productivity

The production capacity per minute, $C$, is given by the following formula:

6 $$\begin{aligned} C = \frac{N}{T} \end{aligned}$$

where $N$ denotes the number of tasks processed, which corresponds to the point at which the AGV arrives at the destination of a given task. Additionally, $T$ represents the time taken for processing, measured in minutes. In this study, time was measured every 5 minutes during continuous experimentation, as explained in Fig. 4.

#### Time to solution

To evaluate the process time improvement, we compute the Time to Solution ($TTS$) for each instance using a multi-solver approach, defined as follows:

7 $$\begin{aligned} \textit{TTS}(\textit{p}) = \frac{t_c \cdot \log (1 - p)}{\log (1 - P_0)}, \end{aligned}$$

where $t_c$ is the actual computational time per output sample, and $p$ is the predetermined precision to reach the ground state. The $TTS$ serves as an indicator of the solver’s performance in a stochastic setting. We set $p = 0.99$ , and $P_0 = 1.0$. The results are summarized in Figs. 9, 11, and 13.

**Figure 8:**
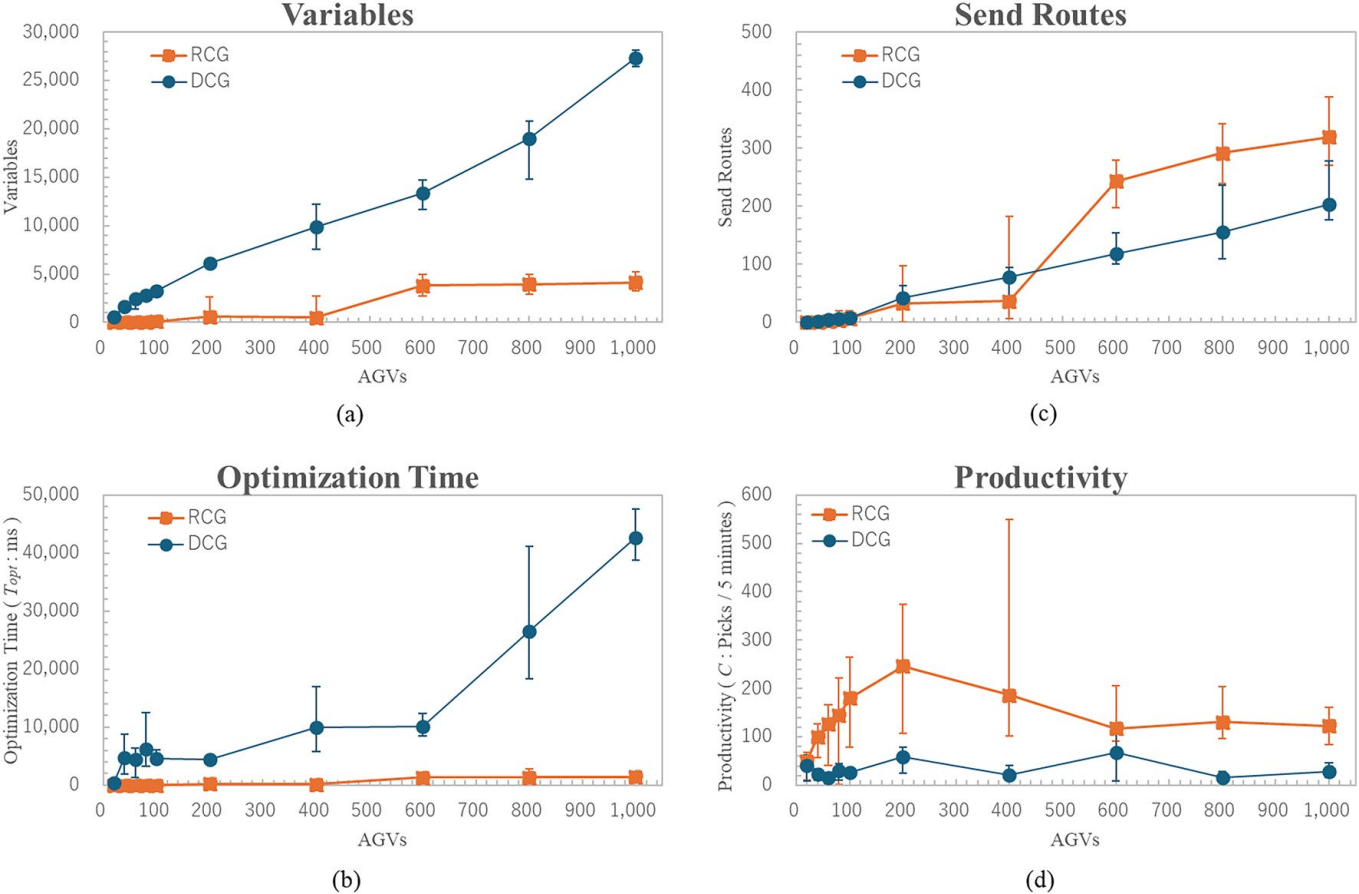
Comparison of optimal route results. The horizontal axis represents the number of AGVs, the vertical axis represents the variables as problem size in (**a**), the optimization time in (**b**), the send routes in (**c**), and the productivity per 5 minutes in (**d**). The results show that the proposed methods help reduce the total number of variables and improve the safety of the optimal processing time, improve the quality of routes and the productivity.The error bars indicated standard error (SE) across repeated experiments. Statistical significance of pairwise comparisons was assessed using Welch’s two-tailed t-test ($\alpha = 0.05$).

**Figure 9:**
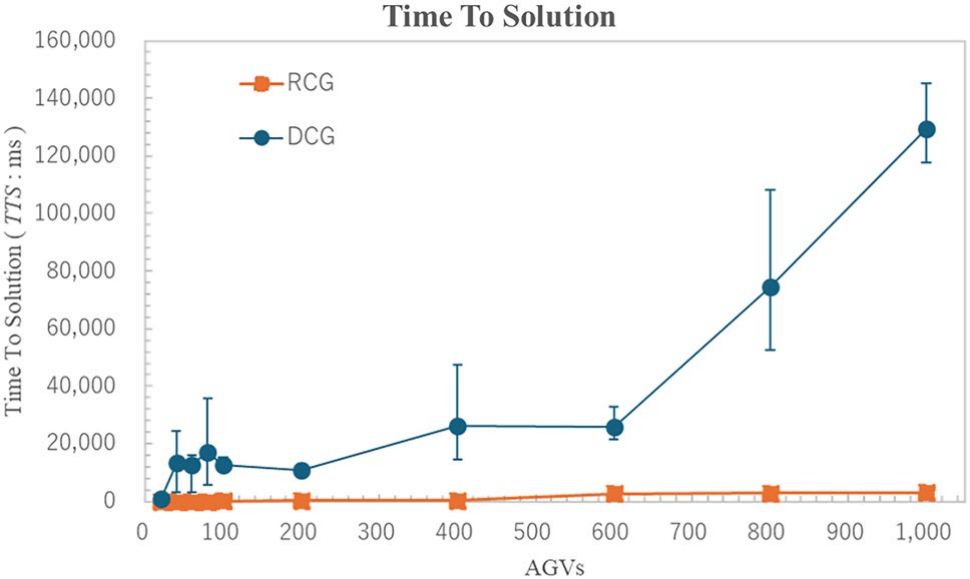
Comparison of time to solution. The horizontal axis represents the number of AGVs, and the vertical axis represents the Time To Solution. It can be seen that the proposed method is able to reduce the overall variables, and therefore, Time To Solution can also be significantly reduced. The error bars indicated standard error (SE) across repeated experiments. Statistical significance of pairwise comparisons was assessed using Welch’s two-tailed t-test ($\alpha = 0.05$).

## Discussion

In this section, we present and discuss the results of the serial experiments described in the previous section.

### Experiment results on a real AGV and a commercial AOS

The results of the demonstration experiment as shown in Table 5, confirmed that the system operated correctly as designed. The real AGV integration scenario shows that there are no conflict resolutions events on the proposal cost function. As shown in the results, small-scale AGVs can achieve route optimization within 2.5 s using both classical and quantum annealing solvers. Locally executed solvers, such as Gurobi and OpenJij_SA, obtain optimal solutions in a very short time. In contrast, solvers that require cloud network access, such as SQBM+ and D-Wave machines, take longer for the optimization process. This is likely due to both solver characteristics and network latency in small-scale scenarios. Regarding route candidates, the proposed method achieved an average reduction of 15% in optimization time and 12% in total processing time compared to existing methods. This improvement is attributed to the proposed method’s consideration of real-time information and a reduction of approximately 26% in the number of qubits used.

### Comparison of the route candidate generation methods

#### Optimal route results

Figure 8a shows the comparison of variables. The horizontal axis was experimented in units of 100 units, from 100 to 1000 units, and on a small scale of less than 100 units. (a) vertical axis shows variables, (b) vertical axis shows optimization time, (c) vertical axis shows number of routes sent to AGVs, and (d) vertical axis shows throughput (Productivity) in 5 minutes increments. In this study, systems with fewer than 500 units are classified as small to medium scale, while those with 500 to 1000 units are considered large-scale. As shown in Fig. 8a, the proposed method reduces the total number of variables by an average of 96% for small-scale or medium-scale systems, and 78% for large-scale systems. Regarding optimization time, as illustrated in Fig. 8b, the proposed method achieves an average reduction of 98% for small-scale or medium-scale cases and 93% for large-scale cases. With respect to the number of optimal routes, as shown in Fig. 8c, the existing method generates, on average, 65% more optimal routes for small-scale or medium-scale cases, whereas the proposed method produces 83% more optimal routes for large-scale cases. This suggests that as problem size increases, the existing method leads to a higher number of violations, while the proposed method yields more effective candidate routes, as further confirmed by the number of route violations. Regarding throughput, as depicted in Fig. 8d, the proposed method enhances throughput by an average of 73% for small-scale or medium-scale problems and 69% for large-scale problems.

#### Time to solution

Figure 9 shows the Time To Solution ${TTS}$. The horizontal axis represents the number of AGVs, and the vertical axis represents the Time To Solution. It can be seen that the proposed method is able to reduce the overall variables, and therefore, Time To Solution can also be significantly reduced. Figures 8 and 9 provide additional statistical analysis based on standard deviations relative to the mean values. Even for the same number of AGVs, the problem variables and complexity can be substantial. As a result, the error bars remain large in some cases. Further investigation in future benchmark optimization experiments is necessary to better understand these variations.

### Comparison of the scalable clustered optimization methods

#### Optimal route results

**Figure 10:**
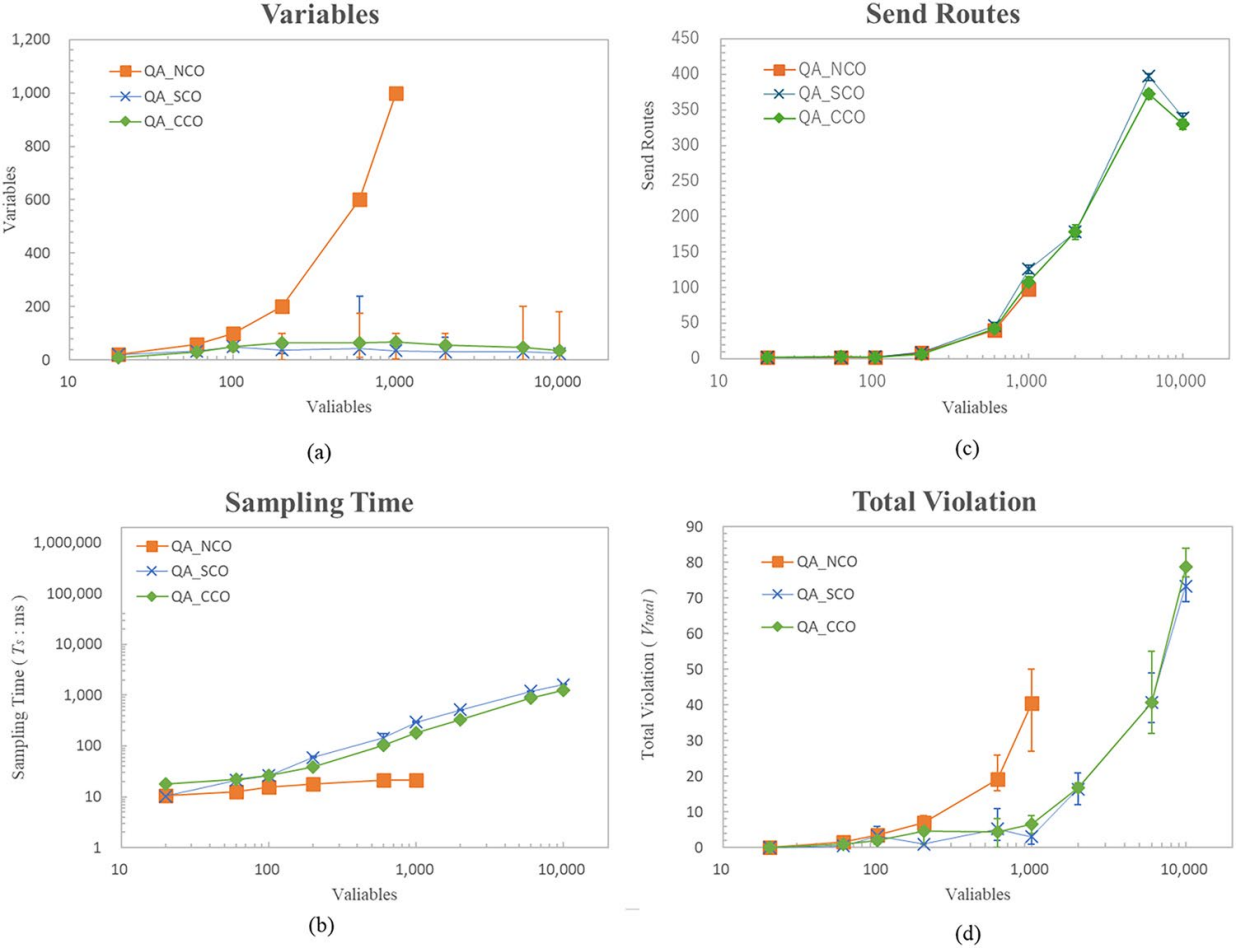
Comparison of the optimal route results. The QA methods in (**a**) and (**b**) demonstrate that, as the problem size increases, a complexity-based problem formulation approach, as opposed to a simple size-based approach, leads to a reduction in sampling time by approximately 28.2% for large-scale problems with 1000 to 10,000 variables. The error bars indicated standard error (SE) across repeated experiments. Statistical significance of pairwise comparisons was assessed using Welch’s two-tailed t-test ($\alpha = 0.05$)..

As shown in Fig. 10a,b, when the QA solver does not use clustering methods, it becomes unable to solve problems with approximately 1000 variables, leading to deviations from the optimal solution and a higher number of constraint violations. In contrast, when clustering methods were applied, problems with up to 10,000 variables could be solved. This was due to the division of the problem into many smaller subproblems. Furthermore, after clustering, it was found that using a complexity-based problem formulation approach, rather than a simple size-based approach, resulted in a reduction of sampling time by approximately 28.2% for large-scale problems with 1000 to 10,000 variables. Furthermore, as shown in Fig. 10c,d, there was no significant difference between the size-based problem generation method and the complexity-based problem generation method. This is likely because the variables and complexity levels of the test problems were relatively low. The percentage of standard deviation with respect to the mean is relatively small for Sampling Time, which is considered one of the key performance indicators. The average percentages of standard deviation for the methods NCO, SCO, and CCO are 5.9%, 5.9%, and 5.0%, respectively.

#### Time to solution

**Figure 11:**
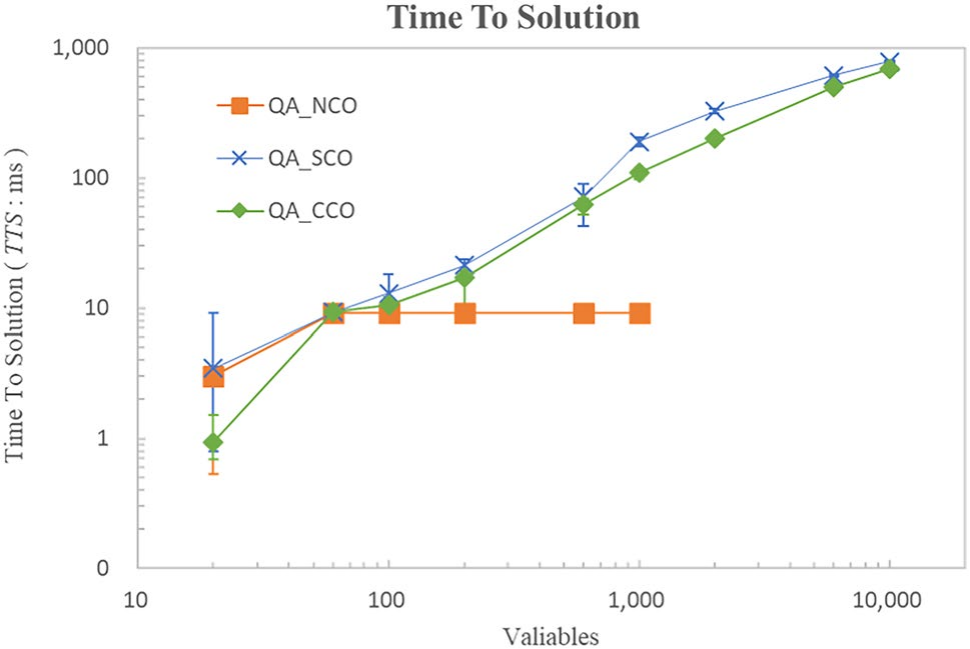
Comparison of the $TTS$. As the problem size increases, there is a tendency for the $TTS$ to increase. Furthermore, for problems with variables ranging from 1000 to 10,000, the complexity-based method reduced the $TTS$ by approximately 27.7% compared to the size-based method. The error bars indicated standard error (SE) across repeated experiments. Statistical significance of pairwise comparisons was assessed using Welch’s two-tailed t-test ($\alpha = 0.05$)..

Figure 11 shows the results of Time To Solution ($TTS$). As the problem size increases, there is a tendency for the $TTS$ to increase. Furthermore, for problems with variables ranging from 1000 to 10,000, the complexity-based method reduced the $TTS$ by approximately 27.7% compared to the size-based method. That leads us to evaluate the complexity-based method to other solvers and methods in the benchmark optimization experiments. The percentage of standard deviation with respect to the mean is relatively small for Time To Solution, which is considered a critical performance metric. The average percentages of standard deviation for the SCO and CCO methods are 9.2% and 7.6%, respectively.

### Comparison of benchmark optimization results

#### Optimal route results

**Figure 12:**
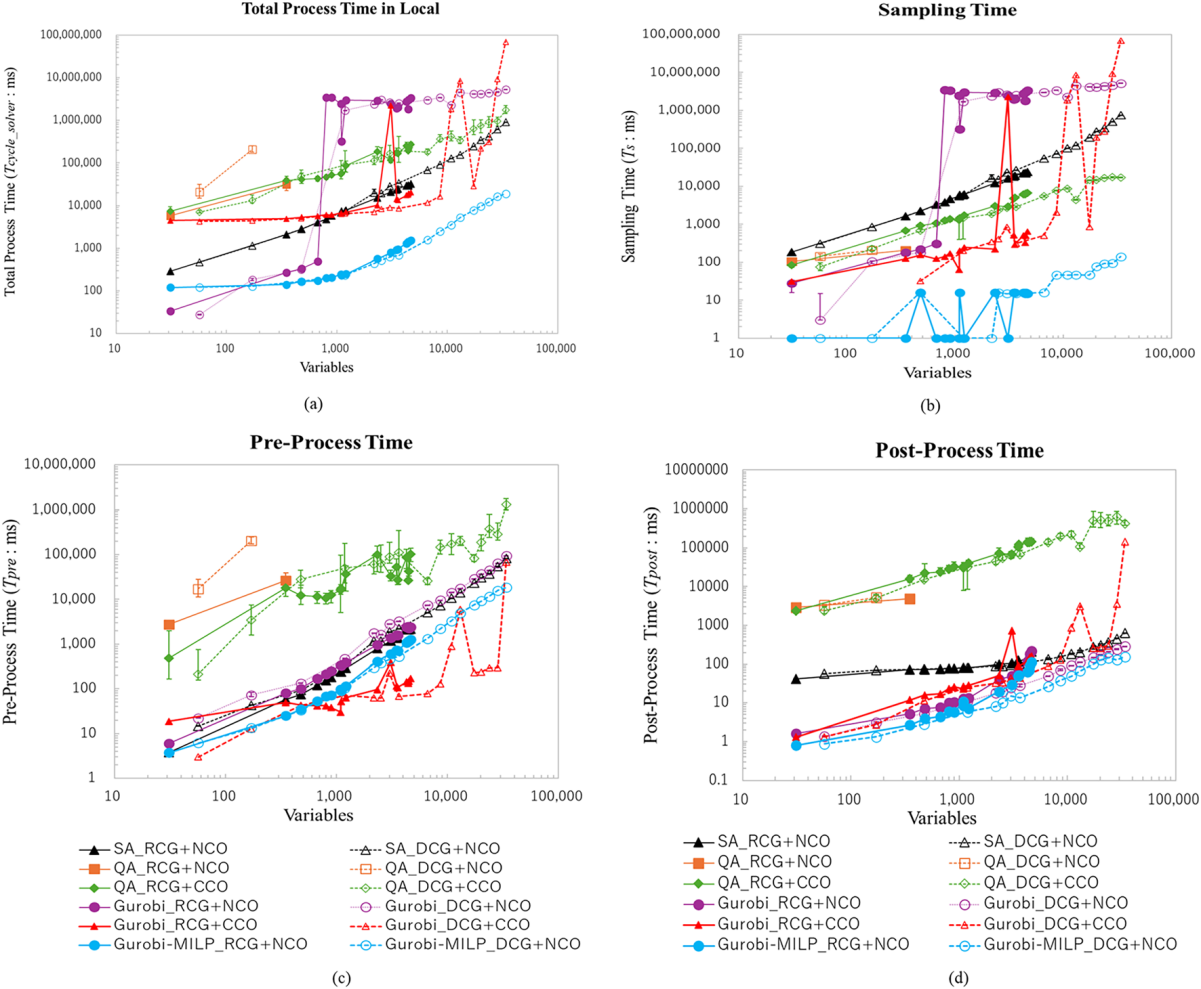
Comparison of the process times. (**a**) Comparison total of process time in solver. (**b**) Comparison of sampling times ($T_s$). (**c**) Pre-process time comparison result. (**d**) Post-process time comparison result. The proposed methods help reduce the number of qubits required, resulting in a maximum sampling time of less than 6.8 s for a problem with 4677 variables as the green diamond line.The error bars indicated standard error (SE) across repeated experiments. Statistical significance of pairwise comparisons was assessed using Welch’s two-tailed t-test ($\alpha = 0.05$)..

The simulation with large scale AGVs with multi solvers scenarios shows that there are no conflict resolutions events on the proposal cost function and the clustering method. Figure 12 shows the comparison of the total process time ($T_{\text {Cycle\_Solver}}$ and $T_{\text {Cycle\_DRD}}$) results. The horizontal axis represents the variables as problem size, and the vertical axis represents the total process time in milliseconds. The total process time was compared for Gurobi, SA, and QA, with and without clustering methods. The results, shown in Fig. 12a,b, represent the total process times in local solvers, and sampling time, respectively. Although the Gurobi-MILP method achieves the overall shortest processing times, this is primarily because the priority in the proposed method is uniformly set to 1. Such a setting could compromise the safety and productivity of AGV operation control, and therefore, should be investigated in future work. When using the Gurobi method with the cost function proposed in this study, the sampling time exceeds 10 s for problems involving more than 1000 variables, and can reach up to 1000 s for problems with 10,000 variables. Simulated Annealing (SA) exhibits similar behavior, with sampling times exceeding 10 s for problems with more than 5000 variables and reaching up to 1000 s for problems with 30,000 variables. On the other hand, QA, without the clustering method, is only shown for problems with fewer than 1000 variables due to the limitations of qubits in the D-Wave machine. The proposed clustering method enables QA to handle large-scale problems, with sizes ranging from 10,000 to 33,822 variables (the green circular line). The proposed clustering method and route candidate generation help reduce the number of qubits required, resulting in a maximum sampling time of less than 6.8 s for a problem with 4,677 variables (the green diamond line). Figure 12c,d shows comparison of the Pre-process time ($T_{\text {pre}}$) and Post-process time ($T_{\text {post}}$). The horizontal axis represents the variables as the size of the problem, and the vertical axis represents the process time in milliseconds. The results for Gurobi_RCG+CCO and Gurobi_DCG+CCO are based on the Gurobi solver. While the processing time was shorter compared to the QA-based methods, the results were less stable. This instability may be due to the presence of highly complex subproblems that still remain after clustering in large-scale problem settings. In contrast, the QA-based methods were evaluated through 15 repeated experiments. For the method (RCG+CCO) using the QA solver, the standard deviations of Sampling Time, Pre-Processing Time, Post-Processing Time, and Total Process Time, expressed as percentages relative to their respective means, were 3.4%, 24.4%, 3.5%, and 4.3%, respectively. For the method (DCG+CCO), these values were 3.8%, 16.3%, 6.7%, and 8.1%, respectively. The elevated variability observed in Pre-Processing Time is primarily attributed to factors such as clustering, partitioning, and communication with the QA machine, which are susceptible to external system environmental conditions. These factors are expected to be optimized in future implementations. Excluding Pre-Processing Time, the standard deviation percentages relative to their respective means for the method (RCG+CCO) and method (DCG+CCO) remained below 5% and 10%, respectively, indicating that the results are stable and statistically reliable.

**Figure 13:**
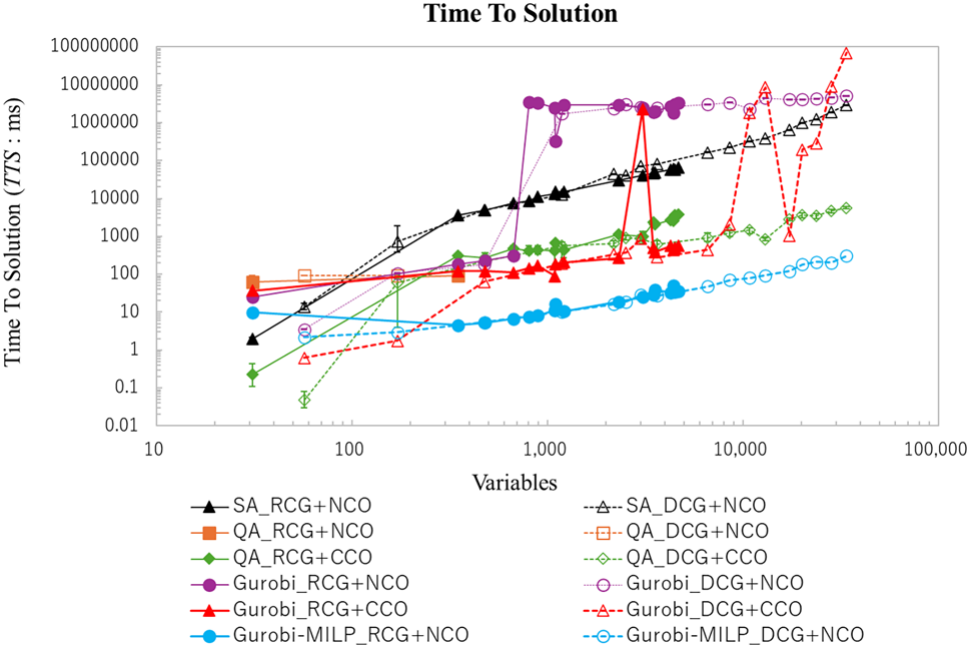
Comparison of time to solution. The horizontal axis represents the variables as problem size, and the vertical axis represents the Time To Solution in microseconds. The proposed method is represented by the green diamond lines, which help reduce the average Time To Solution by 94.2%, compared to the classical SA solver. Although the Gurobi-MILP method yields the overall shortest Time To Solution, this is because the priority in the proposed method is uniformly set to 1. When applying the Gurobi method with the proposed cost function in this study, it achieves results comparable to the proposed method for problems with fewer than 1000 variables. However, it was found that it fails to solve problems with more than 1000 variables.The error bars indicated standard error (SE) across repeated experiments. Statistical significance of pairwise comparisons was assessed using Welch’s two-tailed t-test ($\alpha = 0.05$).

#### Time to solution

Figure 13 shows the comparison of Time To Solution. The horizontal axis represents the variables as problem size, and the vertical axis represents the Time To Solution in microseconds. Although the Gurobi-MILP method yields the overall shortest Time To Solution, this is because the priority in the proposed method is uniformly set to 1. When applying the Gurobi method with the proposed cost function in this study, it achieves results comparable to the proposed method for problems with fewer than 1000 variables. However, it was found that it fails to solve problems with more than 1000 variables. The proposal methods (RCG, CCO) in green lines show the effectiveness of reducing the Time to Solution in almost all variables. For example, Compared to the classical SA solver, the proposal method (RCG+CCO) reduces an average of 94.2% Time To Solution. Furthermore, Compared to the classical SA solver, the proposal method (DCG+CCO) reduces an average of 98.5% Time To Solution. The results for Gurobi_RCG+CCO and Gurobi_DCG+CCO are based on the Gurobi solver. Although the performance difference compared to the QA-based methods was not substantial, the Gurobi-based approaches exhibited instability when applied to large-scale problems. This may be attributed to the possibility that highly complex subproblems still remain even after clustering in large-scale scenarios. The QA-based methods were evaluated through 15 repeated experiments. For the method (RCG+CCO) and the method (DCG+CCO), the standard deviations of the Time To Solution were 4.8% and 7.5% relative to their respective means. Since these standard deviation percentages are below 5% and 10%, respectively, the results are considered stable and statistically reliable.

### Integrated statistical validation

To ensure the statistical validity of the reported performance improvements, formal hypothesis testing was conducted for all experimental results presented in Figs. 8, 9, 10, 11, 12 and 13. All target data satisfied the normality assumption, and thus parametric methods were applied. Pairwise comparisons between the proposed and baseline methods were evaluated using Welch’s two-tailed *t*-test at a significance level of $\alpha = 0.05$.

- **Optimization Efficiency:** For optimization time (Fig. 8b) and time-to-solution (Figs. 9 and 11), the null hypothesis ($H_0$) assumed equal mean values between the compared methods, while the alternative hypothesis ($H_1$) assumed a difference. In all cases, the obtained *p*-values were below 0.001, except for a few configurations in Fig. 11 (AGVs = 20, 100, 200, and 600), where *p*-values exceeded 0.05. These results indicate that the proposed methods (DCG and SCO) achieved significantly shorter optimization and solution times under most conditions.
- **Productivity and Sampling Performance:** For productivity (Fig. 8d) and sampling time (Fig. 10b), similar tests were applied. Although no significant difference was observed for AGVs = 20 and 600 in productivity and for AGVs = 60, 100, and 600 in sampling time ($p > 0.05$), all other cases showed statistically significant differences ($p < 0.01$). This suggests that the performance gains are consistent, except under specific low-load or high-variance conditions.
- **Effect of Clustering Integration:** Comparisons between QA, SA, and QA+Clustering (Figs. 12 and 13) revealed consistent statistical significance across nearly all configurations ($p < 0.001$). QA+Clustering demonstrated significantly shorter process and sampling times than QA alone. Only one exception (variable = 171 in Fig. 13, $p = 0.075$) showed no significant difference, likely due to large variance in both datasets.
- **Summary of Statistical Findings:** Overall, Welch’s *t*-test results confirm that the improvements observed in optimization time, throughput, and sampling efficiency are statistically significant across multiple solvers and experimental settings. The consistency of low *p*-values ($< 0.001$ in most cases) supports the robustness and reproducibility of the proposed approaches. Although formal statistical testing was conducted for completeness, the necessity of such analysis is relatively limited in this context. This is because the AGV route optimization problem is influenced by only a few key factors, primarily the route conditions and priority relationships among AGVs. Furthermore, the solvers used in this study (SA, Gurobi, SQBM+, and QA) are deterministic or well-characterized stochastic algorithms, whose behaviors remain stable under identical experimental conditions.

### Limitations and reproducibility

This study has the following limitations that should be acknowledged. First, the proposed system relies on Sharp’s proprietary AGV Operating System, which is not publicly available for external research use. Second, the number of physical AGVs used for experimental validation was limited; however, large-scale configurations were thoroughly evaluated through extensive simulations. Third, the reproducibility of the results is constrained because only anonymized datasets can be shared. Since we cannot disclose the source code due to industrial confidentiality, the detailed route generation process is provided in Algorithm 2.

## Conclusion and future work

This study proposes a new approach to optimizing the routing of AGVs in large-scale logistics warehouses using Quantum Annealing. As logistics operations expand, efficient AGV routing becomes critical for ensuring safety, reliability, and throughput, particularly in high-density environments. To address the complexity of real-world systems, we introduce an enhanced cost function incorporating a real-time priority factor to improve both safety and routing performance. In this study, we propose a secure and computationally efficient candidate route generation method, as well as an Optimization Problem Clustering technique that decomposes the large problem into smaller, tractable subproblems to reduce computational complexity. The proposed methods are designed to incorporate real-time operational data to ensure collision avoidance, maintain safe operations, and enhance routing efficiency in realistic large-scale warehouse environments.

The effectiveness of the proposed approach on the latest QA machine was validated through performance evaluations comparing classical and quantum-inspired solvers. We validated the algorithm by integrating a small-scale commercial AGV system with a commercial AOS and verifying its connectivity to the classical via local network, to Quantum Annealing machines and Quantum-inspired annealing machine via a cloud network. A simulation framework with 1000 AGVs was employed to assess the practical applicability of the proposed methodology. The results demonstrated that the proposed candidate route generation method reduces the total number of variables by an average of 96% for small-scale or medium-scale systems, and 78% for large-scale systems, while also improving route quality compared to conventional approaches.

Moreover, the clustering method effectively reduced the maximum problem size to below 10,000 qubits, making it possible to control 1000 AGVs while enhancing route quality and safety. While controlling a fleet of 1000 AGVs was previously unfeasible, the proposed method has demonstrated that it is now achievable. Furthermore, after clustering, it was found that using a complexity-based problem formulation approach, rather than a simple size-based approach, resulted in a reduction of sampling time by approximately 28.2% for large-scale problems with 1000 to 10,000 variables.

This research contributes to advancing quantum annealing applications in large-scale logistics operations and presents a promising approach for enhancing AGV route optimization in commercial settings. Experimental results revealed that, for certain large-scale problem instances, processing time was not always sufficient. Future research will focus on refining the pre-processing and post-processing stages to improve computational efficiency. A validate the processing schedule by examining how the order of cluster processing in the proposed clustering method affects acceleration. Additionally, we will compare the proposed clustering method with other clustering methods, such as DBSCAN and t-SNE. Furthermore, we aim to extend the evaluation to continuous optimization problems to further assess the proposed method’s effectiveness in dynamic operational environments. To enhance transparency and reproducibility, we plan to develop open benchmark datasets and modular simulation components, enabling independent validation and benchmarking by other researchers. In addition, we aim to extend our approach to continuous optimization domains and real-time adaptive scheduling, which will allow the system to dynamically respond to operational changes, such as varying AGV priorities and traffic congestion. Ultimately, this research contributes toward the realization of fully autonomous, quantum-accelerated logistics systems that can operate efficiently, safely, and reliably at industrial scale.

## Supplementary Information

Supplementary Information 1.

Supplementary Information 2.

Supplementary Information 3.

Supplementary Information 4.

Supplementary Information 5.

Supplementary Information 6.

## Data Availability

The dataset containing instance features and solver results is available from the corresponding author upon reasonable request.
