# Supplementary material for: Quantum annealing-based route optimization for commercial AGV operating systems in large-scale logistics warehouses
Source: Sci Rep. 2025 Dec 2;15:44047. doi: 10.1038/s41598-025-28481-w (PMC12714809; doi:10.1038/s41598-025-28481-w)
Supplement: Supplementary file 6 — Supplementary Information 6. [file 41598_2025_28481_MOESM6_ESM.zip › Validation_Data/Validation_Data_List.pdf]

Instance ID	Routing Logic	Number of AGVs	Number of Need Route AGVs	Variables size	Experiments Using the Problems
qubo0000001	RCG	20	9	10	
qubo0000002	RCG	20	14	20	Scalable Clustered Optimization Method experiments
qubo0000003	RCG	40	15	30	
qubo0000004	RCG	40	20	40	
qubo0000005	RCG	40	24	50	
qubo0000006	RCG	40	22	60	Scalable Clustered Optimization Method experiments
qubo0000007	RCG	60	38	70	
qubo0000008	RCG	60	38	80	
qubo0000009	RCG	60	35	90	
qubo0000010	RCG	60	41	100	Scalable Clustered Optimization Method experiments
qubo0000011	RCG	80	50	120	
qubo0000012	RCG	100	65	110	
qubo0000013	RCG	100	68	130	
qubo0000014	RCG	100	77	140	
qubo0000015	RCG	100	59	150	
qubo0000016	RCG	100	74	160	
qubo0000017	RCG	200	96	100	
qubo0000018	RCG	200	121	200	Scalable Clustered Optimization Method experiments
qubo0000019	RCG	200	27	300	
qubo0000020	RCG	400	271	400	
qubo0000021	RCG	400	281	503	
qubo0000022	RCG	400	262	600	Scalable Clustered Optimization Method experiments
qubo0000023	RCG	600	453	703	
qubo0000024	RCG	600	475	804	
qubo0000025	RCG	600	480	926	
qubo0000026	RCG	1000	904	999	
qubo0000027	RCG	400	281	503	
qubo0000028	RCG	600	473	999	Scalable Clustered Optimization Method experiments
qubo0000029	RCG	600	273	2092	Scalable Clustered Optimization Method experiments
qubo0000030	RCG	600	509	3001	
qubo0000031	RCG	800	762	3989	
qubo0000032	RCG	800	748	5028	
qubo0000033	RCG	1000	915	5968	Scalable Clustered Optimization Method experiments
qubo0000034	RCG	1000	923	7080	
qubo0000035	RCG	1000	923	8371	
qubo0000036	RCG	1000	645	8627	
qubo0000037	RCG	600	528	8666	
qubo0000038	RCG	1000	926	9161	
qubo0000039	RCG	1000	737	9962	Scalable Clustered Optimization Method experiments
qubo0000040	RCG	1000	909	10323	
qubo0000041	RCG	1000	893	10863	
qubo0000042	RCG	1000	938	11212	
qubo0000043	RCG	800	727	13138	
qubo0000044	RCG	1000	918	13420	
qubo0000045	RCG	1000	933	14153	
qubo1000001	RCG	10	1	2	
qubo1000002	RCG	10	1	9	
qubo1000003	RCG	10	1	31	Benchmark experiments
qubo1000004	RCG	10	2	53	
qubo1000005	RCG	10	3	88	
qubo1000006	RCG	20	2	2	
qubo1000007	RCG	20	3	4	
qubo1000008	RCG	20	10	31	
qubo1000009	RCG	20	9	99	
qubo1000010	RCG	20	11	217	
qubo1000011	RCG	30	14	14	

qubo1000012	RCG	30	18	202	
qubo1000013	RCG	30	16	350	Benchmark experiments
qubo1000014	RCG	30	21	392	
qubo1000015	RCG	30	20	602	
qubo1000016	RCG	40	24	45	
qubo1000017	RCG	40	7	310	
qubo1000018	RCG	40	27	480	Benchmark experiments
qubo1000019	RCG	40	26	600	
qubo1000020	RCG	40	25	782	
qubo1000021	RCG	50	7	310	
qubo1000022	RCG	50	28	532	
qubo1000023	RCG	50	27	669	Benchmark experiments
qubo1000024	RCG	50	37	759	
qubo1000025	RCG	50	33	1034	
qubo1000026	RCG	60	52	459	
qubo1000027	RCG	60	41	700	
qubo1000028	RCG	60	44	804	Benchmark experiments
qubo1000029	RCG	60	42	894	Benchmark experiments
qubo1000030	RCG	60	41	1140	
qubo1000031	RCG	70	51	253	
qubo1000032	RCG	70	42	704	
qubo1000033	RCG	70	52	910	
qubo1000034	RCG	70	45	1051	
qubo1000035	RCG	70	53	1281	
qubo1000036	RCG	80	53	509	
qubo1000037	RCG	80	74	910	
qubo1000038	RCG	80	63	1097	Benchmark experiments
qubo1000039	RCG	80	53	1214	Benchmark experiments
qubo1000040	RCG	80	55	1512	
qubo1000041	RCG	90	76	406	
qubo1000042	RCG	90	57	868	
qubo1000043	RCG	90	60	1095	Benchmark experiments
qubo1000044	RCG	90	61	1262	
qubo1000045	RCG	90	74	1582	
qubo1000046	RCG	100	85	710	
qubo1000047	RCG	100	88	1019	
qubo1000048	RCG	100	68	1281	
qubo1000049	RCG	100	68	1420	
qubo1000050	RCG	100	75	1750	
qubo1000051	RCG	200	171	1492	
qubo1000052	RCG	200	157	2082	
qubo1000053	RCG	200	166	2325	Benchmark experiments
qubo1000054	RCG	200	161	2550	
qubo1000055	RCG	200	165	2947	
qubo1000056	RCG	300	261	2205	
qubo1000057	RCG	300	259	2746	
qubo1000058	RCG	300	257	3077	Benchmark experiments
qubo1000059	RCG	300	264	3303	
qubo1000060	RCG	300	248	3570	
qubo1000061	RCG	400	327	2577	
qubo1000062	RCG	400	360	3228	
qubo1000063	RCG	400	359	3564	Benchmark experiments
qubo1000064	RCG	400	366	3882	
qubo1000065	RCG	400	347	4437	
qubo1000066	RCG	500	420	2326	
qubo1000067	RCG	500	405	2950	
qubo1000068	RCG	500	416	3489	Benchmark experiments

qubo1000069	RCG	500	426	3928	
qubo1000070	RCG	500	434	4650	
qubo1000071	RCG	600	544	3548	
qubo1000072	RCG	600	543	3979	
qubo1000073	RCG	600	554	4284	Benchmark experiments
qubo1000074	RCG	600	552	4650	
qubo1000075	RCG	600	548	5150	
qubo1000076	RCG	700	659	3801	
qubo1000077	RCG	700	653	4169	
qubo1000078	RCG	700	627	4432	Benchmark experiments
qubo1000079	RCG	700	667	4699	
qubo1000080	RCG	700	641	5386	
qubo1000081	RCG	800	745	3976	
qubo1000082	RCG	800	742	4270	
qubo1000083	RCG	800	759	4368	
qubo1000084	RCG	800	765	4611	
qubo1000085	RCG	800	720	5064	
qubo1000086	RCG	900	850	3997	
qubo1000087	RCG	900	853	4275	
qubo1000088	RCG	900	836	4480	Benchmark experiments
qubo1000089	RCG	900	858	4727	
qubo1000090	RCG	900	836	5282	
qubo1000091	RCG	1000	951	4030	
qubo1000092	RCG	1000	953	4425	
qubo1000093	RCG	1000	948	4677	Benchmark experiments
qubo1000094	RCG	1000	952	5036	
qubo1000095	RCG	1000	925	5515	
qubo1000096	DCG	10	1	9	
qubo1000097	DCG	10	1	40	
qubo1000098	DCG	10	1	57	Benchmark experiments
qubo1000099	DCG	10	1	68	
qubo1000100	DCG	10	2	78	
qubo1000101	DCG	20	2	88	
qubo1000102	DCG	20	2	93	
qubo1000103	DCG	20	4	171	Benchmark experiments
qubo1000104	DCG	20	6	337	
qubo1000105	DCG	20	14	595	
qubo1000106	DCG	30	1	112	
qubo1000107	DCG	30	11	347	
qubo1000108	DCG	30	16	476	Benchmark experiments
qubo1000109	DCG	30	17	599	
qubo1000110	DCG	30	18	922	
qubo1000111	DCG	40	17	490	
qubo1000112	DCG	40	27	716	
qubo1000113	DCG	40	25	846	
qubo1000114	DCG	40	29	964	
qubo1000115	DCG	40	27	1207	
qubo1000116	DCG	50	28	679	
qubo1000117	DCG	50	36	984	
qubo1000118	DCG	50	36	1189	Benchmark experiments
qubo1000119	DCG	50	40	1395	
qubo1000120	DCG	50	43	1975	
qubo1000121	DCG	60	40	1274	
qubo1000122	DCG	60	44	1539	
qubo1000123	DCG	60	42	1676	
qubo1000124	DCG	60	49	1859	
qubo1000125	DCG	60	50	2309	

qubo1000126	DCG	70	31	1606	
qubo1000127	DCG	70	51	1923	
qubo1000128	DCG	70	51	2181	Benchmark experiments
qubo1000129	DCG	70	61	2525	
qubo1000130	DCG	70	66	3332	
qubo1000131	DCG	80	57	2090	
qubo1000132	DCG	80	65	2383	
qubo1000133	DCG	80	67	2522	Benchmark experiments
qubo1000134	DCG	80	65	2725	
qubo1000135	DCG	80	75	3058	
qubo1000136	DCG	90	70	2531	
qubo1000137	DCG	90	73	2856	
qubo1000138	DCG	90	78	3006	Benchmark experiments
qubo1000139	DCG	90	74	3181	
qubo1000140	DCG	90	81	3460	
qubo1000141	DCG	100	80	2913	
qubo1000142	DCG	100	84	3147	
qubo1000143	DCG	100	82	3645	Benchmark experiments
qubo1000144	DCG	100	83	3882	
qubo1000145	DCG	100	98	4800	
qubo1000146	DCG	200	173	6123	
qubo1000147	DCG	200	178	6345	
qubo1000148	DCG	200	183	6613	Benchmark experiments
qubo1000149	DCG	200	186	6869	
qubo1000150	DCG	200	193	7316	
qubo1000151	DCG	300	278	7697	
qubo1000152	DCG	300	275	8268	
qubo1000153	DCG	300	280	8584	Benchmark experiments
qubo1000154	DCG	300	282	8962	
qubo1000155	DCG	300	284	9561	
qubo1000156	DCG	400	374	7516	
qubo1000157	DCG	400	364	9428	
qubo1000158	DCG	400	369	10848	Benchmark experiments
qubo1000159	DCG	400	375	11534	
qubo1000160	DCG	400	387	13461	
qubo1000161	DCG	500	461	8508	
qubo1000162	DCG	500	464	11030	
qubo1000163	DCG	500	472	13038	Benchmark experiments
qubo1000164	DCG	500	481	14647	
qubo1000165	DCG	500	477	15759	
qubo1000166	DCG	600	564	12754	
qubo1000167	DCG	600	557	15553	
qubo1000168	DCG	600	581	17360	Benchmark experiments
qubo1000169	DCG	600	576	18203	
qubo1000170	DCG	600	580	18774	
qubo1000171	DCG	700	654	16522	
qubo1000172	DCG	700	658	18297	
qubo1000173	DCG	700	677	20181	Benchmark experiments
qubo1000174	DCG	700	675	22026	
qubo1000175	DCG	700	677	22631	
qubo1000176	DCG	800	749	20026	
qubo1000177	DCG	800	767	22352	
qubo1000178	DCG	800	784	23861	Benchmark experiments
qubo1000179	DCG	800	775	24767	
qubo1000180	DCG	800	773	25580	
qubo1000181	DCG	900	870	24323	
qubo1000182	DCG	900	869	26758	

qubo1000183	DCG	900	880	28471	Benchmark experiments
qubo1000184	DCG	900	874	30400	
qubo1000185	DCG	900	879	31926	
qubo1000186	DCG	1000	981	29594	
qubo1000187	DCG	1000	940	31531	
qubo1000188	DCG	1000	962	33822	Benchmark experiments
qubo1000189	DCG	1000	975	34537	
qubo1000190	DCG	1000	974	35378	
