# Supplementary material for: Quantum annealing-based route optimization for commercial AGV operating systems in large-scale logistics warehouses
Source: Sci Rep. 2025 Dec 2;15:44047. doi: 10.1038/s41598-025-28481-w (PMC12714809; doi:10.1038/s41598-025-28481-w)
Supplement: Supplementary file 6 — Supplementary Information 6. [file 41598_2025_28481_MOESM6_ESM.zip › Validation_Data/Validation_Data_Specification.pdf]

Validation Data Specification

(Rev.1.0)

2025/11/6

SHARP

Contents

1. Problem Classification 2
2. Validation Data Generation Conditions 3
3. Data Structure 4
4. Data List 7

1. Problem Classification

Table 1: Problem Classifiction

**Problem Type**	**Problems No**	**Experiments Using the Problems**
1	qubo0******	Scalable Clustered Optimization Method experiments
2	qubo1******	Benchmark experiments

2. Validation Data Generation Conditions

The AGVs were controlled under the following conditions, generating 45 problems for Problem type 1, of which 9 problems were used in the Scalable Clustered Optimization Method experiments. For Problem type 2, 190 problems were generated, and 34 of those were used in the Benchmark experiments.

Table 2: Generation Conditions

**No**	**Category**	**Parameter**	**Setting value (Problem type1)**	**Setting value (Problem type2)**
1	solver	Solver	Openjij-SA	Openjij-SA
2	Emvioronment	AGVs (*N*)	20,40,…,100,<br>200,400,…,1000	10,20,…,100,200,…1000
3	Emvioronment	MAP	AGVs <=100→<br>150AGVs MAP<br>AGVs>=200→<br>1000AGVs MAP	1000AGVs MAP
4	Input	Orders	1 order/1 AGV	1 order/1 AGV
5	Routing	Candidate routing method	RCG	DCG/RCG
6	cluster	Problem Clustering method	NCO	NCO
7	Routing	Search Time Window (T_max)	300,000,000	300,000,000
8	Routing	Max Candidate Search	20	100
9	Routing	Used_tag_penalty	15	15
10	Routing	Max_tags	18	18
11	Routing	Max_remain_tags	9	10
12	solver	Samples	100	100
13	solver	Sweeps	100	100
14	solver	Lamda 1	300	300
15	solver	Lamda 2	1	1
16	Routing	Penalty On One Tag	TRUE	TRUE

3. Data Structure

The evaluation problems consist of three files: Candidate.csv, MoveTime.csv, and Priority.csv. The meanings of the columns in each file are shown in Table 3. Additionally, examples of each file are presented in Table 4, Table 5, and Table 6.

Table 3: Meaning of the Columns

No	CSV File	Column	Details
1	Candidate.csv	cycleid	Number of the route optimization cycle
2	Candidate.csv	agvid	Number of the AGV
3	Candidate.csv	routeid	Number of the route of each AGV
4	Candidate.csv	route	Details of the route represented by a comma-separated list of tag numbers
5	MoveTime.csv	movetimeij	Evaluation value of the route based on travel time
6	Priority.csv	priority	Priority of the AGV

Table 4: Example of Candidate.csv

cycleid	agvid	routeid	route
146	1	1	105
146	2	1	76
146	3	1	49,20,19,18
146	7	1	652,651,659,667,696
146	8	1	275,304,347,390,433,476,519
146	9	1	388,431,474,517,560,603,625,633
146	10	1	562,605,627,635,643
146	11	1	140,169,198,227,235,243,251,259,267
146	13	1	665
146	15	1	268,276,305,348,391,434,477,520,563
146	15	2	268,276,305,348,391,434,477,520,563,606
146	15	3	268,276,305,348,391,434,477,520,563,606,628
146	15	4	268,276,305,348,391,434,477,520,563,606,628,636
146	15	5	268,276,305,348,391,434,477,520,563,606,628,636,644
146	16	1	172,173
146	17	1	299
146	17	2	299,342
146	19	1	240,232,203,202,201,200

146	20	1	228,236,244,252
146	20	2	228,236,244,252,260

Table 5: Example of MoveTime.csv

cycleid	agvid	routeid	movetimeij
146	1	1	11
146	2	1	15
146	3	1	19
146	7	1	9
146	8	1	26
146	9	1	33
146	10	1	28
146	11	1	25
146	13	1	24
146	15	1	28
146	15	2	27
146	15	3	26
146	15	4	25
146	15	5	24
146	16	1	40
146	17	1	11
146	17	2	16
146	19	1	43
146	20	1	10
146	20	2	9

Table 6: Example of Priority.csv

cycleid	agvid	priority
146	11	1
146	15	2
146	9	3
146	8	4
146	7	5
146	20	6
146	3	7

146	16	8
146	10	9
146	19	10
146	17	11
146	1	12
146	2	13
146	13	14

4. Data List

Please refer to the attached file "Validation_Data_List.pdf"
